# Platelets sequester extracellular DNA, capturing tumor-derived and free fetal DNA

**DOI:** 10.1126/science.adp3971

**Published:** 2025-08-14

**Authors:** Lauren Murphy, Jeanne Inchauspé, Giampiero Valenzano, Pamela Holland, Nikolaos Sousos, Hayley L Belnoue-Davis, Rong Li, Natalie J. Jooss, Camelia Benlabiod, Eleanor Murphy, Zohar Etzioni, Emelie Shepherd, Lucy Denly, Sujata Biswas, Lin Chen, Jennifer O’Sullivan, Michael P Rimmer, Abdullah O. Khan, Christina Simoglou Karali, Nadia Nasreddin, Ian S. Hitchcock, Milka Koupenova, Skirmantas Kriaucionis, Jim R. Hughes, Eric O’Neill, Manu Vatish, Paul Rees, Simon Leedham, Michael Desborough, Adam J. Mead, Benjamin Schuster-Böckler, Christopher D. Gregory, Bethan Psaila

**Affiliations:** 1 MRC Molecular Haematology Unit, MRC Weatherall Institute of Molecular Medicine, Radcliffe Department of Medicine and National Institute of Health Research (NIHR) Oxford Biomedical Research Centre, University of Oxford, Oxford, U.K. OX3 9DS; 2 Ludwig Institute for Cancer Research, Nuffield Department of Medicine, University of Oxford, Oxford, U.K. OX3 7DQ; 3 Department of Oncology, University of Oxford, Old Road Campus Research Building, Roosevelt Drive, Oxford, U.K. OX3 7DQ; 4 Centre for Inflammation Research, Institute for Regeneration and Repair, University of Edinburgh, Edinburgh BioQuarter, 4-5 Little France Drive, Edinburgh, U.K. EH16 4UU; 5 Oxford University Hospitals NHS Foundation Trust, Oxford, U.K. OX3 9DS; 6 Wellcome Centre for Human Genetics, University of Oxford, Oxford, U.K. OX3 7BN; 7 York Biomedical Research Institute and Department of Biology, University of York, Heslington, York, U.K. YO10 5DD; 8 Division of Cardiovascular Medicine, Department of Medicine, University of Massachusetts Chan Medical School, 368 Plantation Street, Worcester, Massachusetts, U.S.A. MA 01605; 9 Nuffield Department of Women’s and Reproductive Health, University of Oxford, John Radcliffe Hospital, Oxford, U.K. OX3 9DU; 10 Department of Biomedical Engineering, Faculty of Science and Engineering, Swansea University, Swansea, Wales, U.K. SA1 8EN; 11 Imaging Platform, Broad Institute of MIT and Harvard, Cambridge, MA,U.S.A

## Abstract

Platelets are anucleate blood cells vital for hemostasis and immunity. During cell death and aberrant mitosis, nucleated cells release DNA, resulting in ‘cell free’ DNA in plasma (cfDNA). An excess of cfDNA is deleterious. Given their ability to internalize pathogen-derived nucleic acids, we hypothesized that platelets may also clear endogenous cfDNA. We found that despite lacking a nucleus, platelets contained a repertoire of DNA fragments mapping across the nuclear genome. We detected fetal DNA in maternal platelets and cancer-derived DNA in platelets from patients with pre-malignant and cancerous lesions. As current liquid biopsy approaches utilize platelet-depleted plasma, important genetic information contained within platelets is being missed. This study establishes a physiological role for platelets that has not previously been highlighted, with broad translational relevance.

Platelets are fundamental to hemostasis, vascular maintenance and both innate and adaptive immunity. Platelets inherit RNAs from their parent megakaryocytes together with translational and splicing machinery. As part of their role in immunity, platelets sense pathogen-derived nucleic acids and internalize DNA and RNA viruses, triggering inflammatory responses (1–4). Intriguingly, in addition to pathogen-derived nucleic acids, RNAs originating from solid tumor cells and from inhaled pine tree pollen have also been detected in human platelets (5, 6) indicating that platelets internalize RNA from a range of cellular sources during peripheral circulation. While platelet RNA has emerging utility as a liquid biopsy approach for hematological (7) and solid malignancies (8, 9), whether platelets contain any form of DNA, and if so, its genomic origin, has not been extensively investigated.

During cell death and aberrant mitosis, nucleated cells release chromosomal DNA, resulting in ‘cell free’ DNA in plasma (cfDNA). Analysis of cfDNA from blood plasma is currently implemented in a wide range of clinical settings, including cancer care (10–16) and prenatal testing (17). A major obstacle to clinical utility is the low sensitivity in scenarios where the abundance of target DNA is low. We hypothesized that in addition to internalizing pathogen-derived exogenous DNA, platelets may clear endogenous cfDNA from plasma, including DNA fragments released by healthy cells as well as those bearing oncogenic mutations. As conventional methods isolate cfDNA from platelet-depleted plasma, any DNA fragments that are sequestered within platelets would be missed. We therefore also hypothesized that including DNA captured by platelets may add value in diagnostic pathways.

## Results

### Platelets contain DNA

**Fig. 1:**
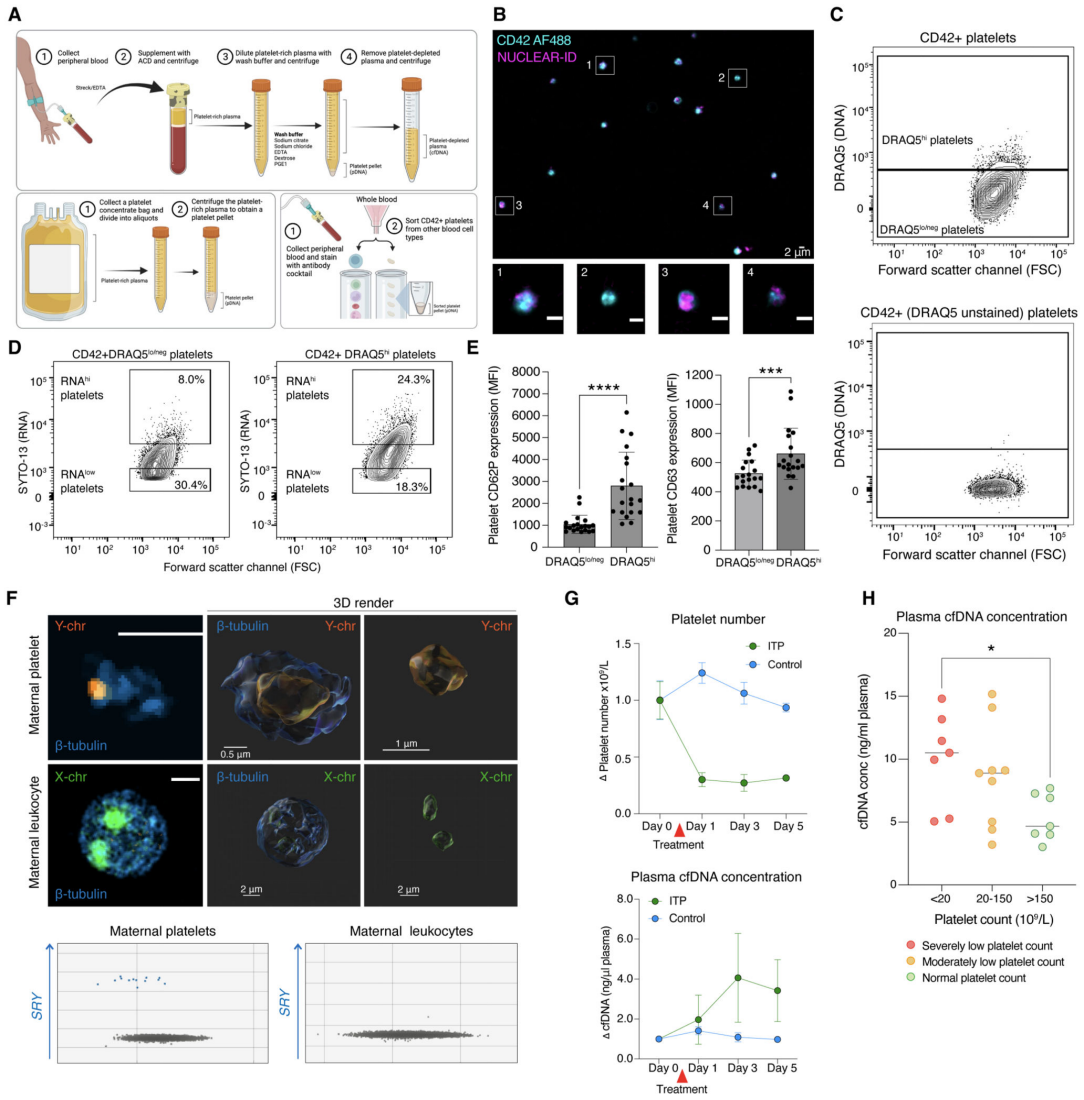
Platelets sequester DNA during circulation. **(A)** Schematic showing sources of purified platelets. Top, sequential centrifugation of peripheral blood to simultaneously extract DNA from a platelet pellet (pDNA) and platelet-depleted plasma (cfDNA); bottom left, centrifugation of healthy donor plateletpheresis concentrates to obtain a platelet pellet; bottom right, fluorescence-activated cell sorting of whole blood. **(B)** Healthy donor-derived platelets identified by CD42 AF488 (cyan) show an internal fluorescent signal for NUCLEAR-ID, a cell permeable dye that intercalates with double stranded DNA (magenta). White boxes show magnified regions, representative images shown, scale bars represent 2 μm. **(C)** Flow cytometric analysis of CD42+ healthy donor-derived platelets stained with and without the DNA marker DRAQ5. Top, 8% of circulating platelets stain strongly positive for DRAQ5, *n* = 20 healthy donors concatenated into a single FCS file. Bottom, single CD42+ stained platelets show no DRAQ5 autofluorescence, *n* = 10 healthy donors, concatenated data shown. **(D)** Flow cytometric analysis quantifying RNA content (SYTO-13 positivity) of CD42+, DRAQ5-hi and DRAQ5-lo/neg platelets (*n* = 10 healthy donors, concatenated data shown). **(E)** Platelets with a higher DNA content (DRAQ5-hi) showed higher levels of activation as determined by P-selectin (CD62P) and CD63 expression. Each dot represents an individual healthy donor. *n* = 19-20 donors for CD63 and CD62P, respectively. Bars show mean ± SD, ***P < 0.001; ****P < 0.0001; as determined by a Wilcoxon signed-rank test. **(F)** FISH and ddPCR showing detection of the Y-chromosome gene *SRY* in maternal platelets, but not leukocytes sampled from mothers of male offspring prior to delivery. Platelets and leukocytes were counterstained with β-tubulin (blue) and imaged using a ZEISS LSM900, 63X magnification. Representative images and ddPCR data shown, 3D rendering performed using ImarisViewer. *n* = 5 female donors (FISH) and *n* = 25 female donors (ddPCR). **(G)** Rise in cfDNA following acute depletion of platelets in healthy mice following administration of an anti-platelet antibody. *n* = 5 mice per day, *n* = 20 mice per condition. Each dot shows mean ± SEM fold change at Day 1, 3 and 5 compared to baseline (Day 0, untreated mice). **(H)** Plasma cfDNA concentrations (ng/ml) for patients with ITP and severely (< 20 x10^9^/L, *n* = 7) or moderately (20 - 150 x10^9^/L, *n =* 9) low platelet counts or counts in the normal range (> 150 x10^9^/L, *n* = 7), *P < 0.05; for a Mann- Whitney *U* test, each point shows data from one individual, with line at the median. ddPCR, droplet digital PCR; FISH, fluorescence *in situ* hybridization; ITP, immune thrombocytopenic purpura; MFI, mean fluorescence intensity; *SRY*, sex-determining region Y.

To determine whether DNA is present within human platelets, platelets were purified from three sources: whole peripheral blood, isolated plasma and ‘plateletpheresis’ concentrates – clinical-grade platelet pools isolated from healthy donors for transfusion purposes (Fig. 1A). We examined platelets by fluorescence microscopy after labeling purified platelets with NUCLEAR-ID Red DNA, a highly specific, cell permeable dye that intercalates with double stranded (ds) DNA. A proportion of platelets showed a clear signal for the DNA probe (Fig. 1B). To verify that this represented nuclear DNA, we confirmed the presence of a nuclear genomic region of the *TP53* gene in platelet DNA (pDNA, fig. S1A & S1B). Immunofluorescence staining of a peripheral blood smear also showed a clear signal for the DNA stain Hoechst in platelets, confirming that the DNA was not acquired by platelets during purification from whole blood (fig. S1C).

To quantify the proportion of platelets that contained DNA we used flow cytometry, examining positivity for DRAQ5, a dsDNA-specific dye. Most platelets showed low-level DRAQ5 positivity while around 8% were strongly positive (DRAQ5^hi^, Fig. 1C & fig. S1D). Prior studies have shown that newly produced or ‘younger’ platelets can be distinguished from ‘older’ platelets by their higher content of RNA, and that younger platelets also have higher expression of markers of platelet receptor activation or degranulation (18–20). Co-staining for RNA and DNA showed that the majority of DRAQ5^hi^ platelets were low or negative for RNA (Fig. 1D, fig. S1E), indicating that DNA is present in both newly produced (RNA-high) platelets as well as in older (RNA-low) platelets. Comparing DRAQ5^hi^ vs. DRAQ5^lo^ platelets showed that DRAQ5^hi^ platelets contained more RNA and had higher expression of markers of activation than DRAQ5^lo/neg^ platelets, implying that the DNA content of platelets also decreased during platelet lifespan and after platelet activation, in parallel with RNA content (Fig. 1D & 1E).

To investigate platelet content of DNA in a larger cohort, we extracted pDNA from peripheral blood of 76 adult donors (table S1 – 4). The extremely high purity of the platelet pellets was confirmed by flow cytometry analysis and cytospin preparations (fig. S1A, S1F & S1G). DNA was obtained from both the platelet pellet and platelet-depleted plasma (cfDNA) in all cases, with a higher yield of DNA from platelet pellets than from matched platelet-depleted plasma (i.e. a conventional cfDNA preparation). In this cohort, a median abundance of 7.18 genomic copies was isolated from platelets per μl of blood plasma, which equates to approximately 0.000012 genomic equivalents of DNA per platelet (fig. S1H).

### Platelets sequester DNA during circulation

To determine whether the DNA detected in platelets was captured during peripheral circulation or solely derived from parent megakaryocytes (21), we collected antenatal blood samples and looked for fragments of the Y-chromosome in platelets of mothers with uncomplicated pregnancies carrying male offspring (table S5). Using Y-chromosome fluorescence *in situ* hybridization, Y-chromosome fragments were visualized in the platelets of mothers carrying a male fetus (Fig. 1F, movie S1). In 100% of samples (*n* = 30), we correctly ascertained the sex of the fetus by the presence or absence of the Y-chromosome gene *SRY* alleles in maternal platelets (Fig. 1F). *SRY* remained detectable in platelets at 24 and 48 hours after delivery of the baby (fig. S1I) and was not detected in mononuclear cells (Fig. 1F) or red blood cells (fig. S1J) from the same blood samples, indicating that free fetal DNA could be detected exclusively in maternal platelets. This suggested that the detection of *SRY* in maternal platelets was not due to ‘contaminating’ cfDNA in the cell pellets, nor the presence of fetal blood cells in the maternal circulation. While cross-placental transfer of fetal platelets cannot be fully excluded, this observation confirmed the presence of nuclear DNA within platelets.

### Platelets ‘clear’ cfDNA from plasma

We reasoned that if platelets were clearing cfDNA from plasma, then the abundance of cell/platelet-free DNA in plasma would increase if the platelet count was reduced. To test this, we induced acute and specific antibody-mediated depletion of platelets in mice. In accordance with our hypothesis, induction of immune thrombocytopenia led to an acute rise in plasma cfDNA concentration (Fig. 1G). Similarly, we found higher concentrations of plasma cfDNA in patients with immune thrombocytopenic purpura (ITP) who had severely low platelet counts (< 20 x 10^9^/L), as compared to those with normal platelet counts (Fig. 1H, table S6, *P < 0.05, *n* = 23).

Together, these data indicate a biological role for platelets in the clearance of cfDNA from plasma.

### Platelets rapidly capture DNA from nucleated cells

**Fig. 2:**
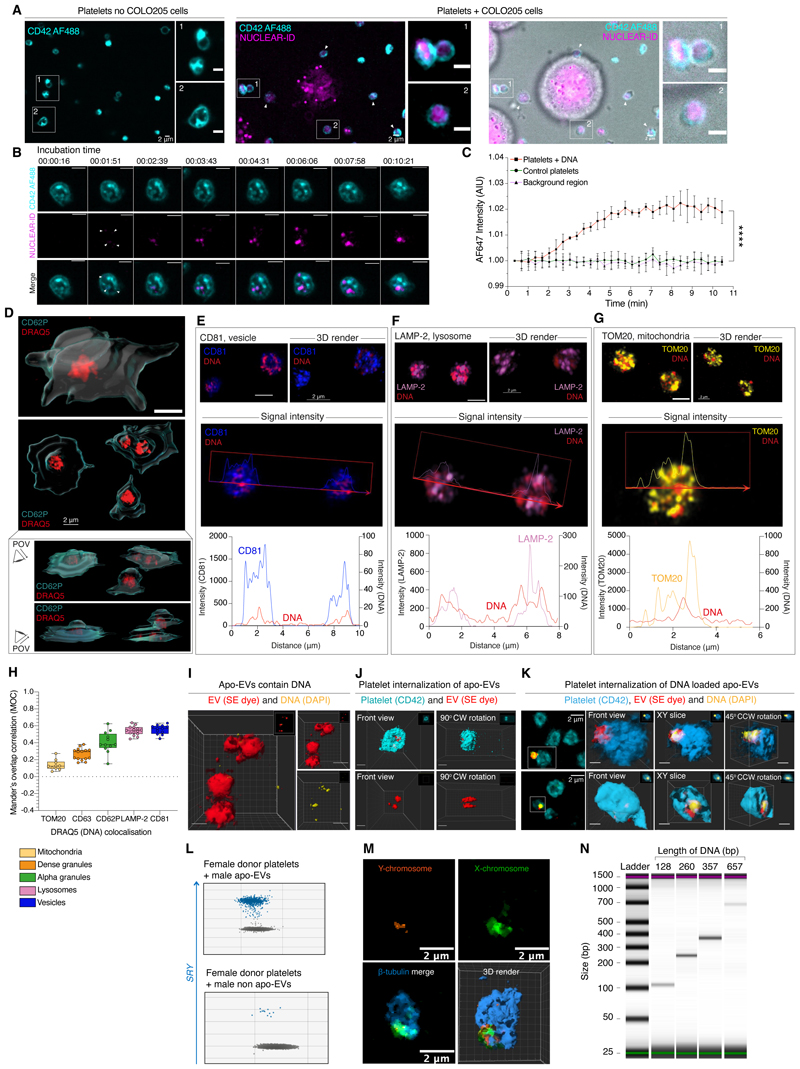
Platelets rapidly internalize DNA released by nucleated cells via uptake of DNA-loaded extracellular vesicles (EVs) and non-membrane encapsulated DNA fragments. **(A)** Healthy donor-derived, CD42 AF488 (cyan)-labeled platelets before (left) and after (middle and right) co-incubation with COLO205 cells labeled with NUCLEAR-ID (magenta) to track DNA transfer. White boxes show magnified regions; arrows highlight platelet uptake of COLO25-derived DNA and scale bars represent 2 μm. Middle (fluorescence) and far right (brightfield) are the same image. **(B)** Live-cell time-lapse imaging of a representative platelet showing internalization of DNA. Scale bars represent 3 μm. **(C)** Time-course quantification of platelet DNA fluorescence intensity, data points show mean ± SD. AF647 signal was measured in background regions to detect fluctuations. ****P < 0.0001; as determined by a Wilcoxon signed-rank test. Tracking shown for platelets incubated with control media (*n* = 168 platelets) or media conditioned by NUCLEAR-ID-labeled COLO205 cells (*n* = 173 platelets). **(D-G)** Representative images acquired using the Airyscan detector of a ZEISS LSM980 and 3D rendering performed using ImarisViewer. **(D)** Healthy donor platelets spread on collagen, stained with CD62P to create a translucent cell surface mask (turquoise) and DRAQ5 (DNA, red). Four representative platelets shown. Bottom panel shows alternate views of the same platelets from above and below, scale bars represent 2 μm. **(E-G)** Top, representative images and 3D renders of two healthy donor platelets showing co-localization of platelet DNA (DRAQ5, red) with **(E)** CD81 (vesicles, blue), **(F)** LAMP-2 (lysosomes, pink), and **(G)** TOM20 (mitochondria, yellow). Bottom, pixel intensity (y-axis) along defined lines (distance in μm, x-axis) was analyzed using ZEISS Zen software. **(H)** Manders’ correlation coefficients were calculated for DNA with mitochondria (TOM20), dense granules (CD63), alpha granules (CD62P), lysosomes (LAMP-2), and vesicles (CD81). Box plots show the median, Q1, and Q3, with whiskers extending to minimum and maximum values. Platelets analyzed per marker: TOM20 (*n* = 10), CD63 (*n* = 15), CD62P (*n* = 12), LAMP-2 (*n* = 15), and CD81 (*n* = 12). **(I)** A representative 3D reconstruction of EV clusters released by apoptotic BL2 cells labeled with an amine-reactive membrane dye (CF658, red) and DNA stain (DAPI, yellow), revealing internalized DNA. Imaged using a ZEISS LSM900, 63X magnification. Scale bars represent 2 μm. **(J)** Platelets stained with CD42 AF488 (cyan) following incubation with labeled EVs (red), showing three EVs within a single representative platelet. 3D modeling shows 90° clockwise rotation. Scale bar represents 1.4 μm. **(K)** 3D reconstruction showing platelet internalization of DNA-loaded apo-EVs (DNA: yellow, EVs: red). Two representative platelets are shown: one with surface-bound DNA-loaded EVs (top) and one with internalized EVs (bottom). Scale bars represent 0.5 μm unless stated otherwise. **(L)** ddPCR quantification of the Y-chromosome gene *SRY* in DNA derived from platelets of a female donor after incubation with apo-EVs and non-apo EVs. Representative data from *n* = 3 independent experiments. **(M)** Representative FISH and 3D render demonstrating fragments of X- and Y- chromosomes in female donor platelets following incubation with male HEL cells. Platelets counterstained with β-tubulin (blue). Imaged using a ZEISS LSM900, 63X magnification and 3D render created using ImarisViewer. **(N)** Electrophoresis data showing recovery of 128-657 bp synthetic DNA fragments from washed healthy donor platelets after overnight incubation. Representative data from *n* = 5 independent experiments. AIU, arbitrary intensity units; apo, apoptotic; BL2, Burkitt’s lymphoma cells; chr, chromosome; CCW, counterclockwise; CW, clockwise; EVs, extracellular vesicles; FISH, fluorescence *in situ* hybridization; POV, perspective of view; SE, succinimidyl ester; *SRY*, sex-determining region Y.

To confirm that platelets sequestered chromosomal fragments released from nucleated cells, we used live cell imaging to visualize DNA uptake *in vitro*. Colorectal adenocarcinoma cells (COLO205) were labeled with DNA probes and washed twice to remove any non-internalized probe prior to co-incubation with platelets. Rapid acquisition of COLO205 cell-derived DNA by platelets was observed (Fig. 2A & 2B, figs. S2A-C, movies S2 & S3). The average DNA fluorescence intensity of the 168 platelets imaged plateaued at ~6 minutes, suggestive of saturation of DNA uptake (Fig. 2C).

### DNA is internalized and released on platelet activation

To determine the localization of platelet DNA, we performed high-resolution Airyscan imaging of healthy donor platelets spread on collagen-coated glass coverslips. During spreading, platelets evaginate the membranes of their open canalicular system (OCS) - an internal surface-connected system of channels - in order to increase their surface area (22). This process extrudes materials captured within the OCS and pushes intracellular contents to the center (22). Imaging of spread platelets co-stained for P-selectin (CD62P) - a marker of platelet activation and de-granulation - and DRAQ5 revealed that DNA was located at the center of spread platelets (Fig. 2D). Calculation of the DRAQ5 staining intensity during progressive computational erosion of the CD62P mask from the edge of the platelet to the center further confirmed that DNA was internalized within the body of the platelet and not held within the OCS (fig. S2D).

We next performed co-localization analyses by co-staining platelets for DNA (DRAQ5), the different types of platelet storage granules (alpha and dense granules), and other organelle markers. We observed strong co-localization between DNA and membrane-bound internal vesicles (CD81 and LAMP-2), with no correlation with mitochondria (TOM20, Fig. 2E-H), confirming that the DNA content of platelets was largely contained within membrane-bound vesicles, and not stored in granules or restricted to mitochondrial DNA.

### Platelets internalize DNA-loaded extracellular vesicles (EVs)

DNA-loaded EVs are released during cellular apoptosis, and viable cells also remove excess cytoplasmic DNA created during aberrant cycles of mitosis by the release of DNA-loaded EVs (23–27). Platelets internalize EVs through their OCS and endocytosis (28), and platelet uptake of tumor cell-derived EVs has previously been shown to transfer cancer cell-derived protein cargo and mRNA transcripts (29).

We therefore investigated whether EVs may mediate DNA transfer to platelets. Platelets isolated from female donors were incubated with EVs derived from male lymphoma BL2 cells or isogenic BL2 cells engineered to over express the apoptosis-suppressing gene *BCL2* (BL2-Bcl-2 cells). This system enabled us to also explore whether DNA-loading of EVs occurred during apoptosis. EVs were isolated from BL2 and BL2-Bcl-2 cells after UV irradiation, generating apoptotic EVs (apo-EVs) from BL2 cells or non-apoptotic (non-apo EVs) from BL2-Bcl-2 cells. The EVs were treated with DNase to remove any non-internalized DNA (Fig. 2I). DNA-loaded EVs were visible adherent to, as well as within, platelets following co-incubation (Fig. 2J & 2K, movies S4 & S5, fig. S2E & S2F). Copies of *SRY* were detectable in platelets from female donors only following incubation with BL2-derived EVs, and in higher abundance following co-incubation with apo-EVs than with non-apo EVs (Fig. 2L & fig. S2G). Similarly, using whole chromosome X and Y FISH probes, fragments of both X- and Y-chromosomes were visualized in female platelets following co-incubation with HEL cells that also derive from a male donor (Fig. 2M).

### Platelets internalize membrane-free DNA fragments

To test whether platelets could also internalize ‘free’ DNA fragments that were not contained within EVs, we exposed healthy donor platelets to synthetic DNA fragments ranging from 128 - 657 base pair (bp) lengths. Platelets efficiently internalized all tested DNA fragments, confirming that platelets sequestered non-membrane bound DNA of varying lengths in addition to DNA-loaded EVs (Fig. 2N).

### Pharmacological modulation of DNA trafficking

**Fig. 3:**
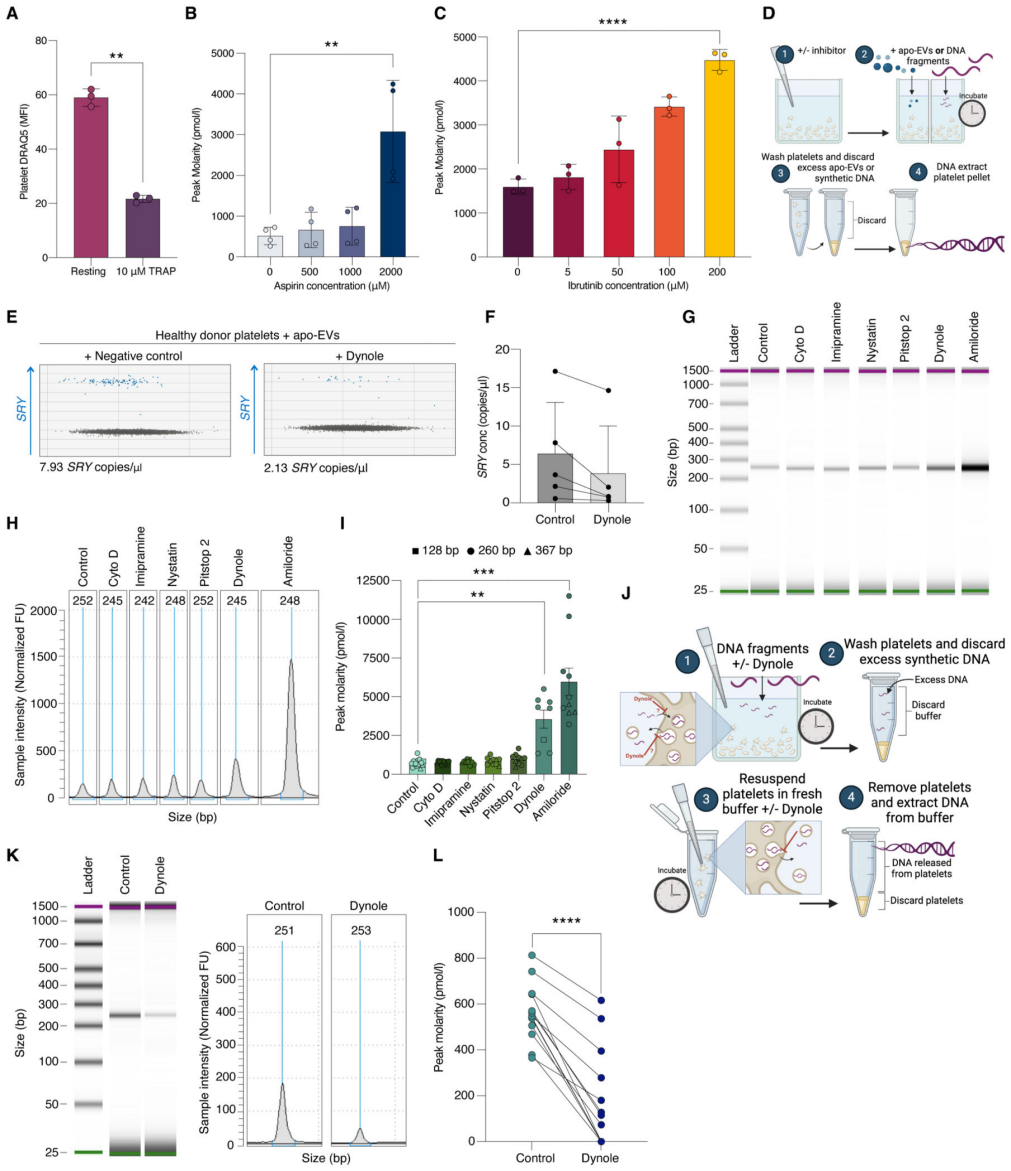
Platelet DNA content can be pharmacologically modulated. **(A)** Flow cytometric analysis of DNA (DRAQ5) content in healthy donor-derived platelets, with and without activation using 10 μM TRAP-6. *n* = 3, points represent data from individual healthy donors, bars show mean ± SD, **P < 0.01; as determined by a paired *t* test. **(B, C)** Inhibition of platelet activation with **(B)** aspirin or **(C)** ibrutinib increases the yield of DNA from platelet pellets. *n* = 3-4 independent experiments, plots show mean ± SD peak molarity corresponding to DNA TapeStation peaks for synthetic DNA fragments co-incubated with platelets prior to washing and DNA extraction. **P < 0.01; ****P < 0.0001; as determined by an unpaired *t* test. **(D)** Schematic of the method for investigating DNA uptake. Platelets were pre-treated with inhibitors, incubated with DNA-loaded apoptotic BL2-derived EVs (apo-EVs) or synthetic DNA fragments, then washed, pelleted, and subjected to DNA extraction. **(E, F)** ddPCR quantification of *SRY* in platelet-derived DNA from female donors after incubation of platelets with apo-EVs treated with or without Dynole. Fewer *SRY* copies were detected in dynamin-inhibited platelets compared to controls. **(E)** Representative ddPCR plot and **(F)** *n* = 5 independent experiments, paired line plot shows mean ± SD, with lines connecting data points before and after inhibition. **(G, H)** Representative electrophoresis showing recovery of 260 bp synthetic DNA fragments from platelets treated with inhibitors known to block components of cargo trafficking. More DNA was extracted from platelets treated with Dynole and amiloride. **(I)** Summary plot showing mean ± SD for all DNA fragment lengths (128-367 bp) following treatment with inhibitors (G, H). Data from four independent experiments (*n* = 4), with 2-5 replicate wells per condition. **P < 0.01; ***P < 0.001; as determined by a paired *t* test. **(J)** Schematic of the method for investigating platelet DNA release. Platelets were treated with or without Dynole, incubated with synthetic DNA, washed, resuspended in fresh buffer, and incubated for 3 hours. DNA was then extracted from platelet-depleted buffer. **(K)** Representative electrophoresis and **(L)** a paired-line plot showing significant inhibition of platelet DNA release following Dynole treatment, with lines connecting data points before and after inhibition. Data from five independent experiments (*n* = 5), with 2-3 replicate wells per condition. ****P < 0.0001; as determined by a paired *t* test. Apo, apoptotic; cyto D, cytochalasin D; ddPCR, droplet digital PCR; EVs, extracellular vesicles; FU, fluorescence units. *SRY*, sex-determining region Y.

To explore whether DNA uptake and release by platelets could be pharmacologically manipulated, we tested the impact of agonists and inhibitors known to block platelet activation and/or specific components of cargo trafficking. Activation of platelets with the canonical platelet agonist thrombin receptor activating peptide (TRAP) decreased their DNA content (Fig. 3A), indicating that DNA is released by platelets upon activation. In keeping with this, the yield of DNA isolated from platelets was higher when platelets were treated with two potent inhibitors of platelet activation - aspirin and ibrutinib, a BTK inhibitor (Fig. 3B-C & fig. S3A).

We next tested a range of inhibitors known to block specific components of cargo trafficking by platelets, including inhibitors of actin (cytochalasin D), membrane ruffling (imipramine), clathrin (Pitstop 2), caveolae-dependent endocytosis (nystatin), dynamin (Dynole) and Na^+^/H^+^ exchange/micropinocytosis (amiloride). In keeping with previous literature suggesting that platelets ‘engulf’ EVs via endocytosis at the OCS membrane, we found that platelet uptake of DNA-loaded apo-EVs was reduced by the dynamin inhibitor Dynole, indicating that DNA-loaded apo-EVs were internalized by clathrin-mediated endocytosis (Fig. 3E-F).

These inhibitors did not reduce the uptake of free DNA fragments by platelets, suggesting that this occurs via an alternative, clathrin-independent mechanism (Fig. 3G). Rather, we found that Dynole and amiloride substantially increased the amount of DNA obtained from the platelets exposed to synthetic DNA fragments (Fig. 3H-I). Dynole and amiloride also block platelet exocytosis and the release of intracellular contents (30, 31). Accordingly, we found that Dynole reduced platelet DNA release (Fig. 3J-L).

Together, these data indicated platelet activation was not required for DNA uptake, but that DNA was released by platelets upon activation. Furthermore, platelet content of DNA can be pharmacologically modulated to increase the abundance of DNA preserved within platelets (fig. S3B).

### Detecting cancer mutations in platelets

Given the growing importance of cfDNA detection in cancer screening and molecular profiling, we sought to determine whether platelets may take-up tumor-derived DNA fragments that conferred cancer-specific genetic variants.

**Fig. 4:**
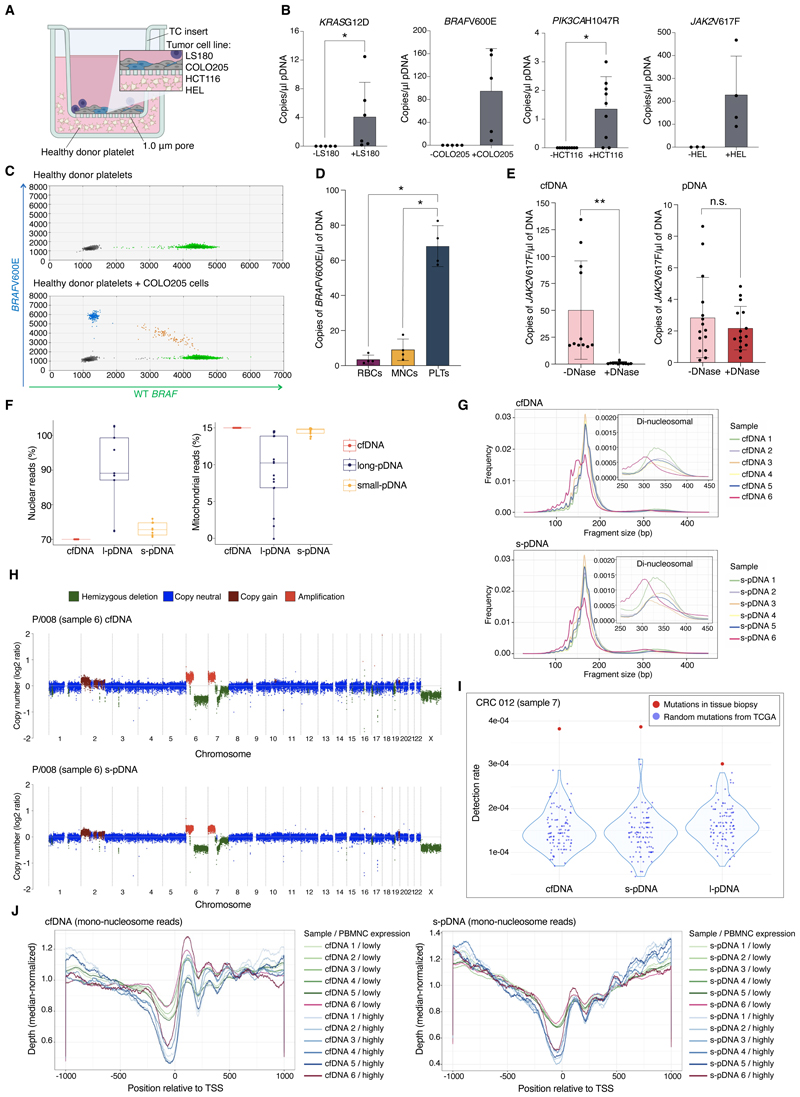
Platelets contain a repertoire of DNA fragments that map over the human nuclear genome, including tumor-derived DNA bearing cancer-associated gene mutations. **(A)** Schematic of the experimental system for co-incubating healthy donor platelets with malignant cells, separated by 1 μm membrane inserts to allow extracellular biomolecule and small EV exchange while preventing cell transfer. After co-incubation, platelets were removed, washed three times, and subjected to DNA extraction. **(B)** Number of copies of mutant alleles detected per µl of DNA extracted from platelets incubated with (+) or without (-) colorectal (LS180, COLO205 and HCT116) and erythroleukemia (HEL) cell lines. Data were collected from one to three independent experiments, with 3-5 replicate wells per condition. Each point represents one co-culture experiment per condition. Replicates per condition were as follows: LS180 (*n* = 2 experiments; 3 replicates each), COLO205 (*n* = 1 experiment; 4 and 5 replicates), HCT116 (*n* = 3 experiments; 3 replicates each), and HEL (*n* = 1 experiment; 3 and 4 replicates). Bars represent mean ± SD, *P < 0.05; as determined by a Wilcoxon paired signed rank test. **(C)** Representative ddPCR analysis showing quantification of wild-type *BRAF* and *BRAF*V600E alleles in pDNA extracted from healthy donor platelets before (top) and after co-incubation with COLO205 colorectal cancer cells (bottom). **(D)** Quantification of *BRAF*V600E in red blood cells, mononuclear cells and platelets following incubation in media conditioned by COLO205 cells. Data are presented as mean ± SD, *n* = 2, individual points represent independent experiments, each with 2 replicates. *P < 0.05; as determined by a Mann- Whitney *U* test. **(E)** Impact of DNase treatment on the detection of *JAK2* mutant alleles in pDNA and cfDNA (media) following co-incubation of healthy donor platelets with HEL cells. Data are presented as mean ± SD, *n* = 5, points represent independent experiments, each with 3 replicates. n.s., not significant; **P < 0.01; determined by a Wilcoxon signed-rank test. **(F)** Percentage of fragments mapping to the nuclear and mitochondrial genomes from cell free DNA, short (> 100 and < 600 bp) and long (> 600 bp) platelet DNA fragments. In the box plot, the center line shows the median, whiskers indicate the minimum and maximum values, and each data point represents one patient, *n* = 15 donors with gastrointestinal carcinoma. **(G)** Fragment length distribution of paired, aligned reads for cfDNA (top) and s-pDNA (bottom). *n* = 6 donors with gastrointestinal carcinoma, samples 1-5 show a primary peak at ~165 bp (mono-nucleosomal) and a smaller secondary peak at ~325 bp (di-nucleosomal, inset). Sample 6 (red line), from a patient with untreated pancreatic adenocarcinoma, has shorter mono- and di-nucleosomal fragments than samples 1-5 (post anti-cancer therapy patients). **(H)** Deviation from median coverage in 100 kbp windows across all chromosomes for cfDNA (top) and s-pDNA (bottom) for P/008 (sample 6), revealing chromosomal aberrations in chromosomes 2, 6 and 7 (copy number gains and amplifications shown in red, deletions in green). **(I)** Detection rates of mutations in cfDNA, s-pDNA and l-pDNA for sample 7 where matched tumor biopsy was available. The red dot indicates mutations detected in the tumor biopsy; blue dots indicate 100 random samples of mutations from other patients with colorectal carcinoma from The Cancer Genome Atlas dataset. **(J)** Read depth distribution around transcription start sites of genes highly (TPM >15, blue) and lowly (TPM <15, green) expressed in peripheral blood mononuclear cells for cfDNA (left) and s-pDNA (right) mononucleosome reads. *n* = 6 donors with gastrointestinal carcinoma, depth per position per sample was normalized to the median read depth across all genes in that expression category. MNC, mononuclear cells; PLTs, platelets; RBC, red blood cells; TCGA, The Cancer Genome Atlas; TPM, transcripts per million; TSS, transcriptional start site; WT, wild type.

Platelets from healthy donors were incubated with a variety of human cancer cell lines harboring common cancer-associated gene mutations, including colorectal cells and erythroleukemia cells (Fig. 4A). Analysis of DNA isolated from platelets following co-incubation with cancer cell lines detected all relevant oncogenic mutations, including *KRAS*G12D, *BRAF*V600E, *PIK3CA*H1047R and *JAK2*V617F (Fig. 4B & 4C). No cancer-specific gene mutations were identified in healthy donor platelets incubated alone, but wild-type alleles were detected (Fig. 4C), indicative of DNA sequestered by platelets prior to blood sampling.

To determine whether the ability to capture extracellular DNA was specific to platelets or shared by other blood cell types, we tested whether *BRAF*V600E derived from COLO205 cells was also sequestered *in vitro* by peripheral blood mononuclear cells (MNCs) and red blood cells (RBCs). While mutant *BRAF (BRAF*V600E) was readily detectable in platelet DNA (pDNA, Fig. 4D), fewer copies of *BRAF*V600E were detected in MNCs and RBCs (Fig. 4D & fig. S3C-E), where they were of negligible abundance in comparison to wild-type alleles (fig. S3D & S3E).

### pDNA is protected from nucleases

To test whether pDNA was protected from external nuclease digestion, conditioned media (analogous to cfDNA) and platelets were treated with DNase following conditioning by HEL cells that carry the *JAK2*V617F mutation. Detection of *JAK2*V617F in HEL cell conditioned media was completely abrogated by DNase treatment (Fig. 4E). In contrast, there was no reduction in the abundance of *JAK2*V617F in platelets following DNase treatment (Fig. 4E), indicating that the majority of DNA was encapsulated by the platelet outer membrane and protected from enzymatic degradation. This was verified using synthetic DNA fragments and target-specific primers (fig. S3F).

### Genomic distribution and origin of pDNA

We then set out to explore the genomic characteristics of pDNA and validate its utility for detection of cancer gene aberrations *in vivo*.

cfDNA has a classical fragmentation profile with a periodicity reflecting nucleosomal footprints (12). Electrophoretic analysis of pDNA showed fragments of around 12’000-16’000 bp, and many samples also showed shorter fragments of around 120-500 bp with nucleosome footprints, similar to the nucleosome footprint of cfDNA (fig. S4A & S4B). Low-input chromatin immunoprecipitation (ChIPmentation) for H3k27 histone acetylation of DNA isolated from platelet pellets and live-cell imaging of platelets co-incubated with H2B-mCherry labeled HEL cells confirmed that the DNA sequestered by platelets was associated with nucleosomes, and that histone modifications are preserved in platelet DNA (fig. S4C & S4D, movie S6).

To compare the genomic origin of pDNA and cfDNA, whole genome sequencing (WGS) was performed on matched pDNA and cfDNA extracted from the peripheral blood of 15 donors, of whom 10 had active malignancy and 5 had undergone surgery and/or chemoradiotherapy, to screen for residual disease (table S4). Size selection was performed on the pDNA to separate the shorter (less than 600 bp) “s-pDNA”, that were similar in length to cfDNA fragments, from the longer fragments (“l-pDNA”).

We performed low pass WGS (mean target coverage of 1x) on s-pDNA, l-pDNA and paired cfDNA (*n* = 15). Platelets contain a small number of mitochondria, estimated at 4-6 mitochondria per cell (32, 33). The majority of fragments from both s- and l-pDNA mapped to the nuclear genome (97.0-99.9% for s-pDNA and 61.7-99.1% for l-pDNA, Fig. 4F). As expected, given that the mitochondrial genome is ~16’500 bp, a higher subfraction (0.9-30.1%) of l-pDNA mapped to the mitochondrial genome, vs. 0.081-2.968% of the shorter fragments (Fig. 4F).

Mapping across the genome revealed that pDNA fragments covered the entire nuclear genome (fig. S4E), mirroring cfDNA and demonstrating that sampling of pDNA would enable genetic variants across the genome to be detected. Compared to cfDNA, the s-pDNA fragments showed more even coverage, as indicated by a lower Gini index (34) (fig. S4F).

### Platelet DNA maps to the nuclear genome, and coverage mirrors cfDNA

We then obtained deeper sequencing (mean sequencing depth 15x ± 3 / 12x ± 1 for cfDNA and s-pDNA, respectively) of paired cfDNA and s-pDNA, including 5 from donors after therapy (samples 1-5), and 2 from donors with active, untreated pancreatic and colorectal carcinoma (table S4). No notable differences were detected in sequencing quality (base quality, 3’ bias, mapping rate) between s-pDNA and cfDNA, except for a higher estimated PCR duplication rate (cfDNA: 3.2% ± 0.6%, pDNA: 11.3% ± 7.2%), indicating lower complexity of short pDNA libraries, presumably due to the size selection.

Nucleosome-bound cfDNA is more protected from degradation than nucleosome-free DNA. This leads to a characteristic fragment-length distribution of cfDNA, with mono- and di-nucleosome length peaks at approximately 167 bp and 320 bp (35). The peak mean fragment length for s-pDNA was 166 bp ± 0.8, with a second, di-nucleosome peak at approximately 328 bp. The di-nucleosome peak was fractionally, but larger in s-pDNA than cfDNA (fig. S5A), likely reflecting the increased protection of pDNA from nuclease digestion, as observed in our *in vitro* experiments (Fig. 4E & fig. S3F).

cfDNA fragments of tumor cell origin are shorter than wild-type fragments (36, 37). In accordance with this, we observed that both mono- and di-nucleosome fragment lengths were notably shorter in both pDNA and cfDNA in sample 6, derived from a patient with an inoperable pancreatic tumor and containing readily detectable tumor-derived cfDNA, indicating that fragmentomic analysis may be applied to platelet DNA to detect the presence of cancer (38), and that platelets capture DNA fragments that resemble those found in cfDNA *in vivo* (Fig. 4G, table S4).

To confirm the capture of circulating tumor cell-derived DNA (ctDNA) by platelets *in vivo*, we used iChorCNA (39) to look for copy number alterations (CNA) and estimate the fraction of tumor-derived DNA. Clonal amplifications and deletions in chromosomes 6 and 7 as well as sub-clonal amplifications in chromosome 2 were detected in both cfDNA and s-pDNA from sample 6 (from the patient with pancreatic cancer), with an estimated tumor fraction of 25% (Fig. 4H). CNAs detected in s-pDNA mirrored those present in cfDNA, and the estimated proportion of tumor-derived DNA was highly correlated between s-pDNA and cfDNA (fig. S5B & S5C). Selecting DNA fragments smaller than 150 bp increased the estimated tumor content of s-pDNA from 25% to 30%, in parallel to the increase seen in cfDNA (fig. S5D). No CNAs were detected in the l-pDNA from sample 6 (fig. S5E), in line with previous reports that ctDNA is enriched in shorter fragments (37), nor in either the cfDNA or pDNA from patients after therapy.

Matched tumor and healthy tissue biopsy samples were available for sample 7 from a patient with colorectal carcinoma, enabling us to look deeper for low-level ctDNA by screening for tumor-specific mutations not present in matched healthy tissue. We compared the detection rate of tumor-specific mutations from the relevant donor with detection of random samples of mutations taken from other patients with colorectal carcinoma (40). The matched tumor mutations were detected at a higher rate than the random mutations in the cfDNA and s-pDNA and at similar frequency (likelihood rate 8.92e-11 for cfDNA and 2.30e-07 for s-pDNA), with lower-level detection in the l-pDNA (*P* <0.0004; Fig. 4I), confirming the presence of ctDNA in platelets.

These data indicated that ctDNA was present within platelets in similar abundance to cfDNA, both in patients with high levels of ctDNA and clear CNAs (>20%, sample 6, Fig. 4H) and in those with extremely low levels (<0.5%, sample 7, Fig. 4I), where targeted analysis and matched tumor tissue was required. Overall, ctDNA was identified in both platelets and cfDNA in 20% of the pre-treatment samples either by detecting CNAs or known tumor mutations, in keeping with the sensitivity of WGS for ctDNA detection reported in large clinical cohorts (41).

The enrichment for nucleosome-bound DNA in cfDNA has previously been used to gain insight into gene expression patterns and cell-of-origin, with prior reports showing that a depletion of reads occurs over transcriptional start sites (TSS) with periodicity in coverage relative to the TSS (42). In addition, DNA methylation studies have shown that the majority (>90%) of cfDNA in both healthy individuals and in those with cancer is of blood cell origin including neutrophils, monocytes, erythroblasts (43) and megakaryocytes (44). We found very similar depletion in sequencing coverage and periodicity around TSS for genes that are highly expressed in peripheral blood MNCs in both pDNA and cfDNA, suggesting a shared cellular origin of pDNA and cfDNA (Fig. 4J & fig. S5F). Notably, the depletion in coverage for high-expression MNC genes was less marked for the sample that had an estimated ctDNA fraction of 25% than for the other samples (Fig. 4J).

To orthogonally validate the cell-of-origin of pDNA, whole genome methylation analysis was performed using the bisulfite-free sequencing method, TET-assisted pyridine borane sequencing (TAPS) (45). The data was then deconvoluted using a published atlas of cell-specific methylation patterns (46). This confirmed a similar cellular origin of pDNA and cfDNA, with the majority originating from blood leukocytes and a minor contribution from hepatocytes. Of note, a higher proportion of l-pDNA than s-pDNA mapped to megakaryocyte-specific loci (35.9% vs. 16.8%), suggesting that more l-pDNA may derive from parent megakaryocytes (fig. S5G).

### High abundance of mutant alleles in platelets in colorectal and pancreatic carcinoma

**Fig. 5:**
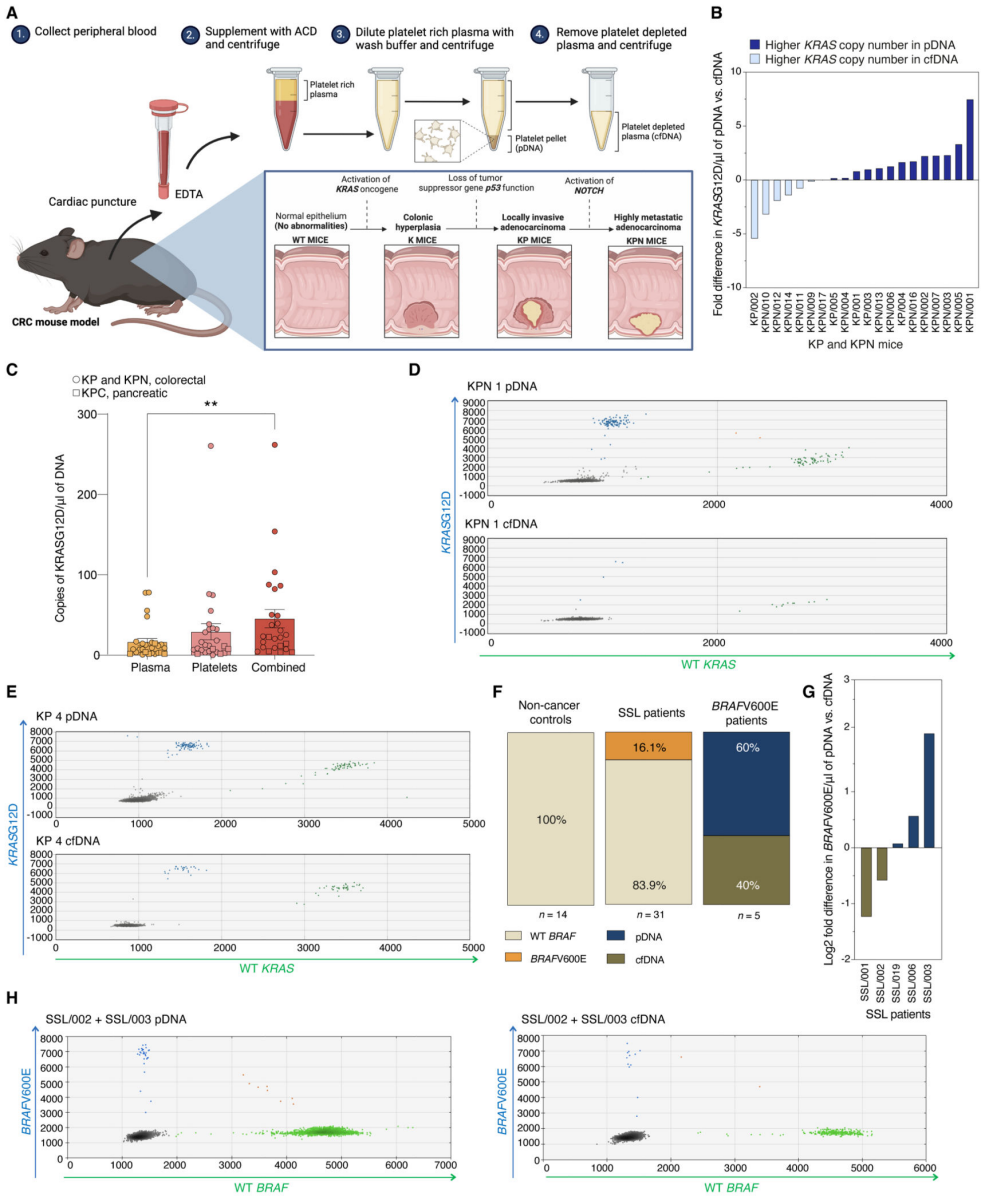
Detection of driver mutations in pDNA and cfDNA from mice with localized and metastatic colorectal adenocarcinoma, and patients with pre-malignant colonic lesions. **(A)** Schematic showing isolation of platelet DNA (pDNA) and cell free (cfDNA) from C57BL/6 mice expressing *KRAS*G12D and *TP53* (KP) mutations via the villin promotor resulting in locally invasive colorectal adenocarcinoma, and mice with *KRAS*G12D, *TP53* and *NOTCH* (KPN) mutations with aggressive, metastatic disease. **(B)** Waterfall plot showing fold difference in copies of *KRAS*G12D detected using ddPCR per μl of DNA extracted from the plasma (cfDNA) and platelets (pDNA) of KP and KPN mice, two weeks after tumor cell implantation, *n* = 5 KP and 15 KPN mice. **(C)** *KRAS*G12D copies detected by ddPCR, shown as copies per µl of DNA extracted from plasma (cfDNA) and platelets (pDNA) of KP, KPN (circle), and KPC (square) mice, compared to the total number of copies from plasma and platelets. *n* = 5 KP, 15 KPN and 6 KPC, datapoints correspond to individual mice, mean ± SEM; **P < 0.01; as determined by a paired *t* test. **(D, E)** Representative ddPCR plots showing *KRAS*G12D (blue) in higher abundance in pDNA (top) than in cfDNA (bottom) in two representative mice, **(D)** a KPN mouse and **(E)** a KP mouse. **(F)** Pie charts showing *BRAF*V600E detection in high-risk colonic lesion patients (SSL, *n* = 31) and colonoscopy-screened controls (*n* = 14). Mutant *BRAF* (>3 positive events) was found in 16.1% (5/31) of SSL patients and 0% (0/14) of controls. **(G)** Relative *BRAF*V600E copy number in pDNA vs. cfDNA for five SSL patients with detectable mutant *BRAF*. Data are log2-transformed with a pseudo count of 1. **(H)** ddPCR plot showing *BRAF*V600E (blue) in pDNA (left) and cfDNA (right) for two representative SSL patients. Data are combined. cfDNA, cell free DNA; CRC, colorectal cancer; ddPCR, droplet digital PCR; pDNA, platelet DNA; SSL, sessile serrated lesions; WT, wild type.

To test whether including an analysis of pDNA would add value to plasma cfDNA in the setting of cancer diagnostics, we used a mouse model of colorectal carcinoma in which expression of *KRAS*G12D and *TP53* mutations are induced via the villin promoter, resulting in locally-invasive colorectal adenocarcinoma with a low incidence of metastases, and mice with *KRAS*G12D^*/+*^, *TP53*^*fl/fl*^ plus *NOTCH*^*fl/+*^, with more aggressive and metastatic disease (47) (Fig. 5A). pDNA and cfDNA were extracted simultaneously from the same blood sample (Fig. 5A).

Mutant *KRAS*G12D as well as wild-type *KRAS* alleles were readily detectable in both cfDNA and platelets of mice with colorectal carcinoma. In over half of the mice (13/20, 65%), copies of *KRAS*G12D per µl of DNA were in higher abundance in pDNA than in cfDNA, both in mice with locally invasive and metastatic disease (Fig. 5B-5E).

To corroborate these findings in a second model and examine an early stage during tumorigenesis prior to tumor dissemination, we used an orthotopic pancreatic cancer model where pancreatic cancer cells were inserted within a Matrigel plug into the body of the pancreas (48). Fourteen days after implantation and prior to local tumor invasion, *KRAS*G12D alleles were readily detected in murine platelets (Fig. 5C & fig. S6A & S6B), and were not reduced in mice receiving low-dose systemic aspirin therapy (fig. S6C).

### *BRAF*V600E in platelets of patients with pre-cancer

Improving the sensitivity for liquid biopsy screening is crucial in the setting of low tumor-burden disease and in patients with pre-malignant lesions, where the abundance of ctDNA is low. Sessile serrated lesions (SSLs) are high-risk, pre-malignant colon polyps driven by mutations in *BRAF* that account for around one quarter of colorectal cancers (49). To explore the utility of pDNA for detecting pre-malignant lesions, we collected platelets and cfDNA from patients found to have SSLs at colonoscopy (*n* = 31) vs. controls (*n* = 14).

Mutant *BRAF*V600E alleles were detected in either the cfDNA and/or pDNA in 16.1% of the patients with serrated polyps (5/31, Fig. 5F), consistent with previous reports of the frequency of detectable ctDNA in SSL cohorts (50, 51). Mirroring our findings in the CRC mouse model, around half (3/5) of the patients with detectable ctDNA had a higher number of *BRAF*V600E copies in pDNA than in cfDNA (Fig. 5F-H, fig. S6D). *BRAF*V600E was detected in none of the pDNA samples from 14 controls (Fig. 5F), demonstrating high specificity of targeted mutational analysis of platelet DNA in this setting and that at least half of the relevant ctDNA is sequestered by blood platelets.

## Discussion

Prior studies of platelet nucleic acid content have only focused on RNA (8, 52–54), although the presence of DNA in platelets has been noted by other groups (3, 4, 21, 55). For example, a study examining the presence of histone proteins in platelets during sepsis showed that 5-35% of platelets were positive for the DNA-specific marker DRAQ5 (55), and the presence of megakaryocyte-derived DNA in platelets was recently corroborated by an independent group (21). Here we have performed a detailed interrogation of platelet DNA content, demonstrating a biological role for platelets that was previously unappreciated – as scavengers of endogenous extracellular cfDNA, including tumor-derived and free fetal DNA.

We show that platelet uptake of DNA can occur by multiple mechanisms – endocytosis of DNA-loaded EVs from apoptotic cells, and uptake of membrane-free DNA fragments. Previous reports indicated that DNA can bind to the platelet surface (56, 57). We found that DNA was also internalized by platelets and held in membrane-bound vesicles, where it was protected from exogenous DNase degradation and subsequently released upon platelet activation. DNA release could be prevented using pharmacological inhibitors, thereby preserving the DNA within platelets where it is protected from degradation, increasing the yield of DNA obtained from platelet pellets.

Several physiological mechanisms exist to limit the abundance of cfDNA, as excess extrachromosomal DNA in the cytosol and in plasma is pro-inflammatory (58, 59). We demonstrated higher and more variable levels of cfDNA in plasma in the setting of severe thrombocytopenia in both a mouse model and in patients. This supports a role for platelets in regulating the cfDNA content of plasma, although we cannot exclude that the higher level of cfDNA observed was due to other consequences of antibody-mediated platelet clearance.

Further work is required to ascertain whether platelets deploy their DNA content at sites of activation. An attractive hypothesis is that platelet clearance may control the non-specific immunostimulatory activity of cfDNA in plasma, and that platelets may release DNA cargo to evoke an immune response in a targeted fashion at sites of platelet activation. Platelets have also been shown to mediate horizontal transfer of RNAs and transcription factors (60), either via uptake of whole platelets by phagocytes, endothelial cells and smooth muscle cells or by the release of platelet-derived microvesicles (59). Whether platelets can similarly act as a vector for the horizontal transfer of DNA fragments, including potentially extrachromosomal DNA (59), and whether platelets modify sequestered DNA prior to release, remains unknown.

Detection and analysis of cfDNA is rapidly being implemented in several clinical settings including cancer screening, molecular profiling, and detection of residual disease as well as in prenatal diagnosis (61–63). Currently, a major limitation is the poor sensitivity for low tumor burden disease. Pre-analytical approaches to increase capture of ctDNA would be of interest. Anucleate red blood cells have also been shown to contain DNA fragments, including cancer-cell derived DNA (64). In our studies, we did not detect *SRY* in red cell pellets of pregnant women and a substantially lower ratio of mutant-to-wildtype alleles was observed in red cells than platelets in our co-culture experiments, suggesting a superiority of platelets over red cells for liquid biopsy-based screening approaches. Our data suggested that a substantial proportion of ctDNA is contained within platelets, and that this information is currently missed from standard sampling protocols that extract cfDNA from platelet-depleted plasma. This study paves the way for validation in larger clinical cohorts to test whether combining cfDNA with pDNA adds value, as well as future research to further establish a role for platelets in cfDNA homeostasis.

## Materials and methods

### Ethical approval, banking and processing of samples

All procedures were performed in accordance with the ethical standards of the Declaration of Helsinki. Patient samples were collected by the Oxford Translational Gastroenterology Unit (TGU) and the Oxford Radcliffe Biobank (ORB). All patients provided informed consent, and procedures were approved by Yorkshire & The Humber - Sheffield Research Ethics Committee (REC reference number: 16/YH/0247), ORB research tissue bank ethics (REC reference 19/SC/0173) and Oxford GI Cohort ethics (REC reference 21\YH\0206).

10 to 20 ml of peripheral blood was drawn into EDTA-coated (Fisher Scientific, cat # 367839) or Cell-Free DNA (Streck, cat # 218997) blood collection tubes from patients presenting with colorectal, esophageal or pancreatic cancer. 10 ml of peripheral blood was collected into Streck tubes, from patients with sessile serrated lesions and from those on the bowel cancer screening pathway or those with inflammatory bowel disease undergoing colonoscopy.

For healthy controls, fully anonymised blood samples were collected from the Oxford Biomedical Research Laboratory under The INForMeD Study (REC reference 16/LO/1376). All donors provided informed consent, and blood was drawn into EDTA-coated tubes. Healthy donor platelet apheresis samples were provided by Oxford NHS Blood and Transplant (NHSBT) through the Non-Clinical Issue (NCI) service.

The usage and collection of healthy human donor blood samples in Edinburgh was authorised under the project “21-EMREC-041 – The Role of Inflammation in Human Immunity”. Freshly drawn blood from healthy human donors was collected into 50 ml BD Falcon tubes containing 4 ml of CPD buffer (16 mM citric acid, 90 mM sodium citrate, 16 mM NaH_2_PO_4_, 142 mM dextrose, pH 7.4).

For obstetric samples, 10 ml of blood was collected from mothers at the time of delivery, and postnatally at 24 and 48 hours after delivery into Streck blood collection tubes. Samples were taken at the Department of Women’s and Reproductive Health at the John Radcliffe Hospital Oxford and approved by the Oxfordshire Research Ethics Committee (07/H0607/74). Sex of the neonate was confirmed after delivery and all mothers provided written informed consent.

Patients with immune thrombocytopenic purpura (ITP) were consented under the INForMeD Study (REC reference 16/LO/1376) and 10 ml of peripheral blood was collected into Streck tubes.

### Animal models

All mice were bred and maintained in accordance with UK Home Office regulations. All experiments were performed under Project Licenses P2FF90EE8, P0B63BC4D and PP6460882 and approved by the University of Oxford Animal Welfare and Ethical Review Body. All mice were housed in individually ventilated cages at the animal unit either at Functional Genetics Facility (Wellcome Centre for Human Genetics, University of Oxford) or the Biomedical Services Facility at the John Radcliffe Hospital (The MRC Weatherall Institute of Molecular Medicine, University of Oxford), in a specific-pathogen-free (SPF) facility, with unrestricted access to food and water, and were not involved in any previous procedures.

For the immune thrombocytopenia model, CD45.2 female, wild-type mice were injected intravenously with either an anti-platelet antibody to reduce platelet count (Emfret, cat # R300) or an IgG control that had no cytotoxic effects on platelets in mice (Emfret, cat # C301). Mice were humanely culled and blood collected via cardiac puncture into EDTA coated microvettes (Sarstedt, cat # 20.1288) at 24, 72 and 120 hours post treatment. Platelet counts were determined using an automated blood cell counter. cfDNA was extracted from plasma and quantified.

For the colorectal cancer (CRC) model, murine blood was drawn from KPN (*villin*Cre^ER^ *Kras*^G12D/+^ *Trp53*^fl/fl^ *R26*^N1icd/+^) and KP (*villin*Cre^ER^ *Kras*^G12D/+^ *Trp53*^fl/fl^) mice via cardiac puncture into EDTA coated microvettes (Sarstedt, cat # 20.1288). All strains used in this model were maintained on C57BL/6J background. Both male and female KP and KPN mice were used.

For the orthotopic pancreatic cancer model, a total of 500 KPC-F cells (passages 9-11) were injected in a volume of 10 µl in a mixture of 50% Matrigel and 50% DMEM (Gibco) using a 0.5 ml Micro-fine insulin syringe (BD). Following anesthesia of the mice using 4% isoflurane/oxygen and clipping of hair, an incision of 1 cm was made in the left abdominal site. The spleen and pancreas were exteriorized using forceps and KPC-F cells were injected in the tail of the pancreas. Successful injection was verified by the appearance of a wheal at the injection site with no leakage through the pancreatic capsule. Spleen and pancreas were gently moved back into the peritoneal cavity; the abdominal wall was sutured with absorbable 4.0 Vicryl suture (Ethicon) and the skin was closed using a 7 mm wound clip. Two weeks post-surgery, after the mice had recovered, blood was drawn via cardiac puncture into EDTA-coated microtainer tubes (BD).

For the cohort of KPC mice treated with aspirin, aspirin (NSC 27223; Selleck Chemicals, cat #. S3017) was administered via intraperitoneal injection on alternate days at a dose of 10 mg/kg. Treatment began on day 4 after tumor cell implantation and continued every other day until day 14. Control animals received intraperitoneal injections of vehicle (2% DMSO in PBS) on the same schedule.

### Blood cell isolation and nucleic acid extraction

Blood tubes were kept at room temperature and handled gently to avoid platelet activation. For human samples, platelets were isolated within 4 or 24 hours of blood sampling (depending on blood tube) using a modified previously published protocol(3). Briefly, whole blood was supplemented with citrate-dextrose solution (ACD) (Sigma Aldrich, cat # C3821-50ML) and centrifuged at 180 x g for 20 minutes at room temperature. Eighty % of the supernatant was removed using a Pasteur pipette, diluted with platelet wash buffer and centrifuged at 600 x g for 20 minutes at room temperature. The resulting platelet pellet was resuspended in pre-warmed HEPES-modified Tyrode’s buffer. The remaining platelet poor plasma was centrifuged at 16’000 x g for 10 minutes at 4°C to ensure the pelleting of all cellular components and the isolation of pure plasma cfDNA. For the collection of platelets from healthy donor platelet apheresis bags, samples were pelleted at 600 x g for 20 minutes at room temperature. Platelet numbers were determined using an automated blood cell counter (Horiba, Pentra ES 60 Cell Counter).

Human mononuclear cells (MNCs) were isolated from freshly drawn venous blood into EDTA coated tubes. Whole blood was supplemented with ACD and centrifuged at 180 x g for 20 minutes at room temperature. The supernatant was removed and the remaining layer diluted with wash buffer (PBS pH 7.4 0.5% FBS, 2mM EDTA). Diluted blood was layered onto density gradient media (GE Healthcare, cat # 5442-03) and centrifuged for 20 minutes at 600 x g, low brake. MNCs were collected from the white inter-phase ‘buffy coat’ using a Pasteur pipette and transferred to a Falcon tube. Human red blood cells (RBCs) were sampled from the upper layer of the RBC/granulocyte pellet and diluted with wash buffer. The remaining RBC/granulocyte pellet was resuspended with chilled RBC Lysis Buffer (eBioscience, cat # 00-4300-54) and incubated at 4°C for 5 minutes. The lysed sample was centrifuged at 1000 x g for 5 minutes, the supernatant discarded and the granulocyte pellet diluted with wash buffer. The remaining white granulocyte pellet was washed, centrifuged and stored at -80°C until required. Purity studies using an automated cell counter provided no evidence of MNC or RBC contamination in the granulocyte pellet.

DNA was extracted from equal volumes of fresh platelets (resuspended in HEPES-modified Tyrode’s buffer) and ‘platelet poor plasma’ using the QIAamp cfDNA kit (Qiagen, cat # 55114), as per the manufacturer’s instructions. Genomic DNA was extracted from nucleated cells using the DNeasy Blood & Tissue Kit (Qiagen, cat # 51104). Extracted DNA was quantified using a Qubit fluorometer (Thermofisher Scientific) and stored at -80°C until required.

Mouse platelets were isolated within four hours of blood collection via cardiac puncture. Whole blood was supplemented with ACD and centrifuged at 100 x g for 10 minutes at room temperature. The plasma was removed and collected into a 1.5 ml Eppendorf tube. The plasma was then diluted with platelet wash buffer and centrifuged at 3500 x g for 11 minutes to pellet the platelets. The platelet pellet was resuspended in pre-warmed HEPES-modified Tyrode’s buffer. The platelet poor plasma was removed and centrifuged at 1300 to 16’000 x g for 10 minutes at 4°C to remove all cells and debris, and the final supernatant collected for cfDNA isolation.

### Cell lines

The following cancer cell lines were used as positive and negative controls for common cancer-associated mutations; HCT116 (CVCL S744) *PIK3CA*H1047R, LS180 (CVCL 0397) *KRAS*G12D, COLO205 (CVCL 0218) *BRAF*V600E, HEL (CVCL_0001) *JAK2*V617F. All cell lines were purchased from American Type Culture Collection (cat # CCL-247, CL-187, CCL-222 and TIB-180 for HCT116, LS180, COLO205 and HEL cells respectively). All cells were cultured according to American Type Culture Collection recommendations and tested monthly for mycoplasma contamination.

NKPCB-NBC12.1F (KPC-F) cells, derived from a KPC mouse (LSL-*Kras*G12D; LSL-*Trp53*R172H; *Pdx1*Cre) were cultured in DMEM (Gibco) supplemented with 10% FBS, 2 mM glutamine, 100 U/ ml penicillin, 100 μg/ ml streptomycin and maintained under at 37 °C with 5% CO_2_.

The human Burkitt’s lymphoma cell line, BL2 (CVCL 1966, cat # CRL-2959) was used to generate tumor cell-derived extracellular vesicles (EVs). Both standard BL2 cells and BL2 cells stably transfected with the apoptosis-suppressing gene *BCL2* were used, to obtain apoptotic (apo-EVs) from BL2 cells and non-apoptotic EVs (non-apo-EVs) from apoptosis-resistant BL2-Bcl-2 cells. Protein expression was regularly tested by flow cytometry, confirming that 98% of the BL2-Bcl-2 cells expressed BCL2. Both cell lines were maintained in 50% X-VIVO medium (50% Gibco RPMI-1640, 50% X-VIVO-20 medium; Lonza, Basel, Switzerland), supplemented with 50 U/ml penicillin and 50 μg/ml streptomycin at 37 °C with 5% CO_2_.

### Generation of extracellular vesicles (EVs) from BL2 and BL2-Bcl-2 cells

BL2 and BL2-Bcl-2 cells were cultured at 20 x 10^6^/ml in 0.1 μm filtered 50% X-vivo 20 and irradiated with ultraviolet light to induce apoptosis with 6 doses at 50mJ/cm^2^, for a total of 300mJ/cm^2^. Apoptosis was monitored hourly by Annexin V and Sytox Blue staining. To isolate EVs, the culture supernatant was centrifuged at 25 x g for 1 hour followed by sequential filtering through a 5 μm mesh filter and a 1.2 μm syringe filter.

### Purification and labeling of apo- and non-apo EVs

The EVs were purified from soluble factors and proteins from the cell culture by size exclusion chromatography using in-house prepared sepharose columns (Thermofisher, cat # 45-000-067 and Sigma Aldrich, cat # CL6B200-100ML). After purification, EV concentration and size profile was measured by Nanoparticle Tracking Analysis using a NanoSight LM10 and Malvern NanoSight software (v3.3). The software analyzes each sample five times to give the average concentration, mean and modal size of each sample. EV size ranged between 150-200 nm and the concentration was between 100-200 x 10^8^/ml. The number of EVs added to platelets was standardized across experiments, using a ratio of 50 x 10^8^ EVs with 1 x 10^7^ healthy donor platelets. For imaging experiments, prior to incubation with platelets, the purified EVs were suspended in 0.1 μm filtered HBSS and were stained with Biotium CF568 NHS ester with a covalent attachment of the dye to EV proteins, according to the manufacturer’s protocol (Biotium, cat # NC1542764). To remove unbound dye, the EVs were purified again by size exclusion chromatography through sepharose columns.

### Flow cytometry and fluorescence-activated cell sorting (FACS)

All experiments were carried out using an LSRFortessa X-20 flow cytometer (Becton, Dickinson and Company). Platelets and paired plasma were prepared as previously described, pelleted and resuspended in HEPES-modified Tyrode’s buffer. The samples were stained with a cocktail of antibodies for 20 minutes at room temperature in the dark, prior to being washed and resuspended in fresh buffer for flow cytometric analysis. The antibodies used are as follows, CD42 FITC, CD41 PE-Cy7, CD3 PE, CD11b and CD33 APC, CD19 APC-Cy7 and CD235a BV650. To assess the nucleic acid content of platelets, healthy donor blood was drawn into EDTA-coated tubes and platelets were isolated as previously described. The platelets were stained with the antibody CD42 PeCy7 and nucleic acid stains DRAQ5 (DNA) and Syto-13 (RNA) for 20 minutes at room temperature in the dark. After incubation, the platelets were washed and resuspended in fresh HEPES-modified Tyrode’s buffer for analysis. Compensation beads (were stained and analyzed in parallel, as an additional negative control to fluorescence minus one (FMO) controls to confirm no non-specific binding of the nucleic acid stains (BD Invitrogen, cat # 01-1111-42). To assess platelet activation, platelets were isolated from healthy donor blood drawn into citrate tubes. Following isolation, platelets were maintained at a physiological concentration of ~300 x 10^6^ /ml.

To evaluate the activation status and DNA content of healthy donor platelets, after isolation, platelets were stained with CD42 FITC, and antibodies for activation; CD63 PB and CD62P BV857. The DNA content was simultaneously assessed by the inclusion of the DNA marker, DRAQ5. After a 20-minute incubation at room temperature, the platelets were washed and resuspended in HEPES-modified Tyrode’s buffer.

To determine changes in platelet DNA content upon activation, 10 µl of platelets were added to Eppendorf tubes containing the antibodies CD42 FITC, CD63 PB and CD62P BV857 and the DNA stain DRAQ5. The platelets were then treated with 3 µl of either a ‘resting’ or an ‘activation’ buffer, both comprising HEPES-modified Tyrode’s buffer supplemented with 20 mM calcium (Sigma-Aldrich, cat. # 21115-100ML). For the activation buffer, the agonist TRAP-6 was added at concentrations of 5, 10, 50, or 100 µM (10x the final concentration, Bachem AG, cat. # 4031274.0025). After a 15-minute incubation at room temperature, the platelets were washed twice prior to resuspension in HEPES-modified Tyrode’s buffer and analyzed.

The BD FACSymphony S6 was used for cell sorting. 30 µl of healthy donor blood was diluted with an antibody cocktail to a final volume of 100 µl. The platelets were incubated for 20 minutes at room temperature in the dark, washed and resuspended in fresh buffer. The staining solution included the following antibodies, CD42 FITC, CD3 BV421, CD11b and CD33 APC, CD19 APC-Cy7 and CD235a FITC. Platelets (CD42^+^, CD3^-^, CD11b^-^, CD33^-^, CD19^-^ and CD235a^-^) were sorted into 15 ml falcon tubes containing HEPES-modified Tyrode’s buffer. The platelets were centrifuged at 600 x g for 20 minutes, the supernatant removed, and pellet frozen at -20°C.

Single color-stained controls and FMO controls were used for all experiments. FACS data were collected using BD FACSDiva software (v9.0) and gates were set using FMO controls and negative populations. All antibodies and nucleic acid stains are listed in table S7.

### Droplet Digital PCR (ddPCR)

ddPCR assays were ordered from Bio-Rad. All ddPCR assays were conducted on a QX200 Droplet Digital PCR System using the manufacturer’s recommended protocol and reagents (Bio-Rad Laboratories). Positive and negative controls were included in every assay. Analysis was performed using the QuantaSoft software (Bio-Rad Laboratories, Watford UK).

To detect mouse *KRAS*G12D, a ddPCR assay was designed for the detection of the *KRAS* p.G12D c.35G>A point mutation. Primers for the detection of *KRAS* were as follows: forward 5’-GCCTGCTGAAAATGACTGAG-3’ and reverse 5’-CGTAGGGTCATACTCATCCAC-3’. Two dual labeled probes were also used to target the wild-type and mutant sequences.

Wild-type 5’-HEX-ACGCC[+A][+C]CAG[+C]TCCAA-BHQ1-3’ and mutant 5’-6FAM-AC[+G]CC[+A][+T][+C]AG[+C]TCCAA-BHQ1-3’. Square brackets indicate LNA bases (Merck Life Sciences).

### Synthetic DNA oligo generation

Single stranded DNA oligos were designed to include the mouse *KRAS*G12D genetic marker and synthesized by Integrated DNA Technologies (IDT, Iowa USA). Synthesized DNA was reconstituted according to the manufacturers protocol. PCR assays for the detection of *KRAS* were performed using the KAPA2G Fast HotStart PCR Kit (Roche, cat # KK5512) and primers were as follows: forward 5’-GCCTGCTGAAAATGACTGAG-3’ and reverse 5’-CGTAGGGTCATACTCATCCAC-3’. Fragmentation profiles of DNA oligos were analyzed using an Agilent Tapestation 2200 system (Agilent Technologies). Electrophoresis data was analyzed using the Tapestation Analysis Software (Agilent).

### In vitro co-culture of platelets, tumor cells, EVs, and synthetic DNA oligos

To explore uptake of DNA shed from tumor cells, platelets (3 x 10^5^ /µl), MNCs (0.075 x 10^5^/µl) and RBCs (3 x 10^5^ /µl) from healthy donors were added to individual wells within a 24-well plate and a 1.0 µm TC Insert (Sartstedt, cat # 83.3932.101) placed into the well. Cancer cells (0.03 x 10^5^/µl) were carefully placed in the inserts and the plate incubated for approximately eight hours at 37°C. Control samples were incubated simultaneously but without the addition of tumor cells. After incubation, the inserts were removed and the blood cells transferred to Eppendorfs. To ensure complete removal of any contaminating cfDNA in the media, all cells/platelets were washed and centrifuged at 600 x g for 10 minutes. The supernatant was removed and the pelleted blood cells re-suspended in fresh buffer. The cells were washed for a total of three times and finally resuspended in 1 ml of fresh pre-warmed HEPES-modified Tyrode’s buffer prior to DNA extraction using the QIAamp cfDNA kit. Following DNA extraction, the samples were quantified and ddPCR was used to identify the presence of mutant alleles.

To investigate platelet uptake of DNA containing EVs, 50 x 10^8^ labelled, purified apo- and non-apo EVs resuspended in in Tyrode’s buffer were incubated with 0.1x 10^8^ platelets for three hours at 37°C with gentle inversion every 30 minutes. Following incubation, the samples were centrifuged for 20 minutes at 800 x g. The supernatant was discarded, and the platelets re-suspended in 300 µl of PBS. EV and platelet pellets were treated with DNase as per manufacturer’s protocol (Sigma Aldrich, cat # AMPD1) and then DNA extracted using the QIAamp cfDNA kit. Following DNA extraction, the samples were quantified and assayed for the male *SRY* gene using ddPCR.

To understand whether platelets also take up ‘free’ DNA fragments, platelets were incubated with synthetic DNA fragments ranging from 128-657 bp. Healthy donor human platelets were aliquoted into Eppendorf tubes and spiked with reconstituted synthetic DNA oligos for a final concentration of 10 ng/ml. The platelets were mixed gently by pipetting and incubated at 37°C overnight. Following incubation, the platelets were centrifuged for 10 minutes at 600 x g. The supernatant (containing excess DNA oligo) was discarded, and the platelets re-suspended in fresh buffer. The platelets were washed for a total of two times and finally resuspended in 1ml of fresh pre-warmed HEPES-modified Tyrode’s buffer prior to DNA extraction using the QIAamp cfDNA kit. Eluted DNA was PCR amplified using the KAPA2G Fast HotStart PCR Kit (Roche, cat # KK5512) and fragment length was confirmed using the Agilent Tapestation.

### DNase treatment to investigate platelet DNA internalization

Platelets were incubated with HEL erythroleukemia cells as previously described. Following co - incubation, platelets were treated with DNase according to the manufacturers protocol (Ambion DNA-free Kit, cat # AM1906). To confirm catalytic activity of the enzyme, DNase was also added to conditioned media. Following DNase treatment, the platelet samples were washed with 500 µl of 5mM EDTA and centrifuged at 600 x g for 10 minutes. The supernatant was removed and the wash repeated. After the second centrifugation, the supernatant was discarded and the platelet pellet resuspended in 1 ml of fresh pre-warmed HEPES-modified Tyrode’s buffer. Following DNase inactivation of the conditioned media, 900 µl of HEPES buffer was added to a final volume of 1 ml in preparation for DNA extraction. For control samples, no DNase was added and 10 µl of 1M tris-HCL was added in replacement of 10X DNase I Buffer for the platelet samples.

In a separate experiment, healthy donor platelets or an equal volume of HEPES-modified Tyrode’s buffer (to mimic plasma) was spiked with a 260 bp synthetic DNA oligo and incubated overnight. The samples were then treated with DNase according to the manufacturers protocol (Life technologies, RapidOut DNA Removal Kit, cat # K2981). Following DNase treatment, the platelet samples were washed and centrifuged at 600 x g for 10 minutes. The supernatant was removed and platelet pellet wash repeated. After the second centrifugation, the supernatant was discarded and the platelet pellet resuspended in 1 ml of fresh pre-warmed HEPES-modified Tyrode’s buffer. For ‘cfDNA’ (buffer) samples, DNase was inactivated and 900 µl of HEPES buffer was added to a final volume of 1 ml. DNA was extracted from all samples using the QIAamp cfDNA kit. Eluted DNA was PCR amplified and the fragment length confirmed as previously described. For control samples, no DNase was added and 10 µl of 1M tris-HCL was added in replacement of 10X DNase I Buffer for the platelet samples.

### Inhibition of cargo-trafficking by platelets

Healthy donor human platelets were treated with the following inhibitors or appropriate negative control molecules; Dynole 34-2 (Abcam, cat # ab120463, 30 µM), Dynole 31-2, Negative control (Abcam, cat # ab120464-5mg, 30 µM), Pitstop (Abcam, cat # ab120687, 25 µM), Pitstop 2 Negative control, (Abcam, cat # ab120688, 25 µM), Cytochalasin D (Santa Cruz Biotechnology, cat # CAS 22144-77-0, 5 µM), Nystatin (Merck Life Science UK, cat # N9150-20ML, 30 units/ml), Amiloride (Abcam cat # ab120281, 500 µM), Imipramine (Abcam, cat # ab141046, 5 µM). After treatment, platelets were spiked with a 260 bp synthetic DNA oligo for a final concentration of 10 ng/ml and incubated at 37°C overnight. Following incubation, the platelets were centrifuged for 10 minutes at 600 x g. The supernatant (containing excess inhibitor/ negative control and DNA oligo) was discarded, and the platelets re-suspended in fresh buffer. The platelets were washed for a total of two times and finally resuspended in 1 ml of fresh pre-warmed HEPES-modified Tyrode’s buffer prior to DNA extraction using the QIAamp cfDNA kit. Eluted DNA was PCR amplified and the fragment length confirmed as previously described.

To assess the impact of platelet activation inhibition on platelet DNA uptake, platelets were treated with 500, 1000 or 2000 µM aspirin (Merck Life Sciences, cat # A2093-500G) or 5, 50, 100 or 200 µM Ibrutinib (PCI-32765) (Stratech Scientific Ltd, cat # S2680-SEL-5mg). Following treatment, a 260 or 367 bp synthetic DNA oligo was added to resting platelets for a final concentration of 10 ng/ml DNA and incubated at 37°C overnight. Following incubation, the platelets were centrifuged for 10 minutes at 600 x g. The supernatant (containing excess inhibitor/ negative control and DNA oligo) was discarded, and the platelets re-suspended in fresh buffer. The platelets were washed for a total of two times and finally resuspended in 1 ml of fresh pre-warmed HEPES-modified Tyrode’s buffer prior to DNA extraction. Eluted DNA was amplified by PCR and the Agilent TapeStation was used to confirm fragment length.

To assess the impact of dynamin inhibition on platelet secretion, platelets were treated with Dynole or paired negative control, exposed to the 260 bp synthetic DNA oligo and incubated overnight. Following incubation, the platelets were pelleted, resuspended in fresh buffer, re-treated with the inhibitor or negative control and incubated for 3 hours at 37°C. After the second incubation, the platelets were pelleted and discarded, and the supernatant (platelet ‘releasate’) was collected. The supernatant was centrifuged at 16’000 x g for 5 minutes to ensure complete removal of any contaminating platelets and subject to DNA extraction, PCR amplification and fragment analysis.

To understand the impact of dynamin inhibition on platelet EV internalization, 0.1x 10^8^ healthy donor platelets were incubated with either the dynamin inhibitor, paired negative control or DMSO for 30 minutes at 37°C. After incubation, 50 x 10^8^ labelled, purified apo-EVs re-suspended in Tyrode’s buffer were added to the treated platelets and incubated as previously described prior to DNase treatment, DNA extraction and *SRY* ddPCR.

### H3k27ac ChIPmentation of platelet chromatin

Platelets were isolated from plateletpheresis samples from 3 independent healthy donors. 13 ml of each sample was centrifuged at 600 x g for 30 minutes at room temperature, the supernatant was removed and platelet pellets were immediately used for H3K27ac ChIPmentation using a protocol tailored for low-DNA input samples from a published protocol(65), using the H3k27ac antibody (Diagenode, cat # C15410196). In brief, bead-bound chromatin undergoes a one-step adaptor insertion via Tn5 tagmentation and only one post-PCR DNA purification is performed to reduce sample loss.

For each reaction, 5 μl A-Dynabeads (Invitrogen cat # 10001D) were coated with the H3K27ac antibody (Diagenode, cat # C15410196) (150 μl PBS, 0.5% BSA, 1x Proteinase inhibitor). The antibody-bound beads were then washed in PBS and 0.5% FBS, before being added to samples for overnight incubation.

Platelet pellets were incubated in 600 μl lysis buffer (50 mM Tris pH 8, 10 mM EDTA, 0.5% SDS, 1x Protease Inhibitor) for 20 minutes on ice. Then, 1% Triton-X100 was added, and the samples centrifuged to pellet insoluble material. The supernatant was split into three 200 μl sub-samples, and the samples were incubated overnight with the H3k27ac antibody coated beads. Before incubation, 5% of the sample was kept aside for input control tagmentation.

After overnight incubation, the beads were washed three times with RIPA wash buffer (50 mM HEPES pH 7.6, 500 mM LiCl1, 1 mM EDTA, 1% NP-40 / IGEPAL CA-630, 0.7% Na-Deoxycholate), followed by one wash with TE and then 10 mM Tris pH8. Beads and input control samples were then tagmented in tagmentation buffer (50 mM Tris pH 8, 25 mM MgCl2, 50% Dimethylformamide) with Tn5 transposase (Illumina, cat # 20034197) for 10 minutes at 37°C and immediately cooled down. Beads were immediately washed. Library preparation was then conducted with Nextera-based sequencing primers(66). qPCR and fragmentation analysis were performed.

### Immunofluorescence microscopy

#### Live

To visualize platelet DNA uptake *in vitro*, washed human platelets were stained with anti-CD42 AF488 (BioLegend, cat # 303914) for 30 minutes at room temperature. Separately, COLO205 cells or H2B-mCherry HEL cells were labeled using either the highly specific live cell DNA stain, SiR-DNA (Spirochrome, cat # CHF260.00), NUCLEAR-ID Red DNA stain or DAPI (HEL only) as per the manufactures’ protocols (Enzo Life Sciences, cat # ENZ-52406). Stained COLO205 or H2B-mCherry HEL cells were then washed and added to collagen type-1 (Corning, cat # 354236)-coated microscopy slides (Greiner Bio-One, cat # 543079). CD42 stained platelets were added to the wells containing tumor cells immediately before imaging. To analyze endogenous wild-type DNA within healthy donor platelets, platelets were co-stained with anti-CD42 AF488 and NUCLEAR-ID Red DNA and imaged alone.

Regarding platelet uptake of tumor derived DNA from conditioned media, COLO205 cells were labeled with NUCLEAR-ID Red. Following staining, the cells were washed with MEM Alpha 5% FCS, pelleted and the supernatant removed. The cells were washed a further two times to ensure complete removal of the DNA stain and resuspended in 200 µl of fresh media. The cells were incubated at 37°C and vortexed periodically to encourage cell death and DNA release. After two hours, the COLO205 cells were centrifuged at 16’000 x g for 10 minutes at 4°C. Supernatant was removed from the pelleted cells, transferred to a fresh Eppendorf and the cell pellet discarded. Healthy donor platelets were stained with anti-CD42 AF488 and added to wells of Poly-L-lysine coated slides (Ibidi, cat # 81201). DNA labeled COLO205 cell conditioned media was added to the platelets immediately before imaging. All live imaging was performed using the Zeiss Spinning Disk Confocal with a 63X oil immersion objective.

#### Fixed

BL2 cell-derived apo-EVs were isolated, labeled with an amine-reactive fluorescent dye (Biotium, cat # 92131) and frozen as previously described. Prior to imaging, the EVs were thawed and co-stained with DAPI for 30 minutes at room temperature. The EVs were washed twice, pelleted at 20’000 x g for 30 minutes at 4°C and resuspended in HBSS buffer. Labeled EVs were then added to Poly-L-lysine coated chamber slides and incubated overnight at 4°C. Following incubation, the supernatant was carefully removed and the EVs fixed with 4% formaldehyde at room temperature for 30 minutes. The fixative was then removed and the EVs gently washed three times with PBS. To confirm imaging of EVs and not auto-fluorescent debris, PBS was stained with DAPI, incubated overnight, and fixed onto slides alongside the EV preparations. The slides were mounted and the EVs and PBS controls resolved by fluorescence microscopy using a Zeiss LSM900 with a 63X oil immersion objective.

To visualize platelet sequestration of EVs, 5 x 10^9^ apo-EVs labeled with an amine-reactive fluorescent dye were thawed and added to 1 ml of 10 x 10^6^ washed healthy donor platelets and incubated for three hours at 37°C. To ensure adequate mixing, the cells were inverted gently every 30 minutes. Following incubation, the cells were centrifuged at 800 x g for 20 minutes, low brake. The supernatant (containing the EVs) was removed, and the platelets resuspended in PBS. Platelets were centrifuged onto a Poly-L-lysine coated chamber slide at 600 x g for 20 minutes and fixed in 2% formaldehyde/PBS for 10 minutes at room temperature. The fixative was removed and the platelets washed twice with PBS. The platelets were then labeled with anti-CD42 AF488 for 20 minutes at room temperature. After labeling, the slides were washed, mounted with ProLong Gold Antifade Mountant (Thermofisher, cat # P36930) and imaged using the Zeiss LSM900.

### Histology, immunohistochemistry, and imaging of peripheral blood films

Cell contamination was assessed after cytospin of the resuspended platelet pellet onto a glass slide (5 minutes at 500 r.pm.) and May–Grünwald Giemsa staining, according to standard protocols. Images were obtained using an Olympus BX60 Microscope.

KPC mice were sacrificed, and pancreatic tissue was taken for p53 immunohistochemical staining. Briefly, a total of 24 hours post fixation in 10% neutral buffered formalin at 4°C, tissues were transferred to 70% ethanol and stored overnight at 4°C. Fixed tissues were processed overnight using the STP120 Spin Tissue Processor (Thermo Fisher Scientific) and embedded the next day in paraffin wax. Tissues were cut into 4 µm sections using a Leica RM215 microtome and adhered onto SuperFrost Ultra Plus Adhesion slides (Thermo Fisher Scientific) before drying overnight in a 37°C incubator. After deparaffinization, rehydration in an ethanol gradient (100%, 70%, 50%), and antigen retrieval with Citrate Buffer Antigen Retriever (Sigma-Aldrich), slides were incubated with Dual Endogenous Enzyme-Blocking Reagent (Dako) for 1 hour, washed in PBS, incubated with 5% goat serum for 30 minutes, washed in PBS again, and incubated with the primary antibody, p53 (Cell Signaling Technologies, cat # 2524) diluted 1:2000 for 2 hours at room temperature. Slides were then washed in PBS with 0.05% Polysorbate 20 and incubated with the secondary antibody, horse anti-mouse IgG (Cell Signaling Technologies, cat # 7076), diluted 1:100 for 45 minutes at room temperature, washed in PBS with 0.05% Polysorbate 20 again, and incubated with 3,3′-Diaminobenzidine (DAB) chromogen for 3 minutes at room temperature. After staining, slides were dehydrated in an ethanol gradient (50% 70%, 100%), followed by tissue clearing in xylene (ThermoFisher). Slides were mounted with dibutylphthalate polystyrene xylene (DPX; Sigma-Aldrich), dried for 24 hours and scanned using an Aperio ScanScope CS digital slide scanner (Leica Biosystems) at 20X magnification.

Healthy donor blood was used to prepare a peripheral blood film. The blood smear was fixed with 100% methanol for 30 seconds and stained with CD42 AF488 (BioLegend, cat # 303914) to identify platelets and the DNA stain Hoechst (Invitrogen, cat # H3570), to identify nucleated cells. The slide was mounted with a coverslip and imaged using the Leica SPE.

### Fluorescence In Situ Hybridization (FISH)

Platelets in suspension were centrifuged onto Poly-L-lysine coated chamber slides and fixed in 2% formaldehyde/PBS for 10 minutes at room temperature. The platelets were then permeabilized in 0.5% Triton X-100/PBS for 10 minutes at room temperature and washed twice in 0.05% Triton-X 100/PBS. The platelets were washed in 0.02% Tween20/PBS (PBST) and incubated with the primary anti-β-tubulin antibody (Sigma Aldrich, cat # T5201) diluted in blocking buffer for 30 minutes at 37ºC in a humid box. Following primary labeling, the platelets were washed with PBST and incubated with the secondary donkey anti-mouse IgG H&L AF405 antibody (Abcam, cat # ab175658) diluted in blocking buffer for 30 minutes at 37ºC. The labeled platelets were then washed with PBST, fixed and washed with PBS for 10 minutes at room temperature. FISH staining was carried out according to the manufacturer’s protocol. Briefly, slides were incubated in 0.1N HCL, washed with 2x SSC and dehydrated in 70% ethanol for 3 minutes. Equal volumes of X- and Y-chromosome probes (Metasystems, cat # D-0323-050-FI and D-0324-100-OR) were added to the slide and covered with a 22 x 22 mm^2^ coverslip. The slides were incubated at 85ºC for 5 minutes, sealed with rubber cement and incubated in a humidified chamber at 37°C overnight. Following hybridization, the rubber seal was removed, and the slides washed in 0.1x SSC at 60ºC. Slides were mounted and imaged using the Zeiss LSM900 with a 63X oil immersion objective. Control cells (HEL cells and MNCs) were fixed onto slides, permeabilized and labeled with the X- and Y- chromosome paints. Following overnight hybridization, the cells were washed and counterstained with DAPI.

### Airyscan imaging of platelet ultrastructure

Glass coverslips (VWR, cat # 631-0150) were coated with 5 µg/ml fibrillar collagen-I (Horm), (Nycomed) and incubated at 4ºC overnight in a 24 well plate. The coverslips were blocked with 5 mg/ml BSA for 1 hour at room temperature. After blocking, the BSA was removed and 300 µl of washed healthy donor platelets diluted to 1x10^7^/ ml was added to the wells. The platelets were left to spread for 45 minutes in a humidified chamber at 37°C. Following incubation, the liquid was carefully removed and washed with PBS. The platelets were fixed with 5% formalin for 10 minutes at room temperature, washed with PBS and permeabilized with 0.1% Triton-X 100/PBS (Sigma, cat # SLCF5969) for 5 minutes. The slides were washed with PBS and blocked for 30 minutes with blocking buffer (1% BSA + 2% goat + 2% donkey serum). The primary antibodies: mouse anti- human CD63, mouse anti- human LAMP2, rabbit anti-human TOM20, rabbit anti- human CD81 and mouse anti- human CD62P were diluted in blocking buffer and incubated with the platelets at 4ºC overnight. Following primary incubation, the coverslips were washed three times with PBS and incubated with the DNA stains (DRAQ5, NUCLEAR-ID Red, Hoechst) and the secondary antibodies; goat anti- mouse IgG H&L AF568 and goat anti- rabbit IgG H&L AF488 for 45 minutes at 37°C. Following incubation, the coverslips were washed and mounted onto imaging slides using Hydromount (Scientific Laboratory Supplies Ltd, cat # NAT1324). The slides were imaged using the Airyscan function of a Zeiss LSM 980 confocal microscope (63X objective lens, NA 1.4).

### Quantitative analysis of imaging data and image analysis software

Images were analyzed using ImageJ (v2.1.0) (National Institute of Health, US-MD) and 3D reconstructions were generated in Imaris Viewer v9.7.0 (Oxford Instruments, Abingdon). CellProfiler (v4.0.7) (Broad Institute, US-MA) was used to quantitatively analyze platelet-tumor cell interaction. In brief, cells were segmented using the ‘Identify Primary/Secondary Object’ module. Thresholding strategies were employed to accurately mask cells for segmentation. Platelets were tracked using the ‘Track Objects’ module. Finally, AF647 intensity was measured using ‘Measure Object Intensity’ and data ‘Exported to Spreadsheet’. This image-analysis pipeline, as well as others can be recreated in Cell Profiler tool (freely available from the Broad Institute at www.cellprofiler.org).

For co-localization analysis, we calculated the Pearson’s correlation coefficient, r and Mander’s overlap coefficient, *R* for each platelet by manually defining a cuboid region (90 x 90 pixels; 3.8 x 3.8 µm), enclosing fluorescence from all three channels. The equations used to calculate fluorescence correlation coefficients are described in the Supplementary Text, with calculations performed using MATLAB as described by Jung et al(67).

To assess the location of the DNA fluorescence within the platelet we developed a simple algorithm in MATLAB to calculate the total intensity of the DNA marker in a 3- dimensional (3D) platelet mask that was subsequently eroded away from the surface to the center. First, we generated a 3D platelet mask by applying a one level Otsu thresholding algorithm to the CD62P fluorescence signal. The DNA intensity within the platelet was obtained by integrating the DRAQ5 fluorescence signal from pixels within the 3D platelet mask. To determine the position of the DNA fluorescence within the platelet, we used a series of one pixel erosion operations on the original 3D mask. After each erosion step, a convex hull was applied to ensure there were no ‘holes’ in the mask. The integrated DNA fluorescence intensity was then obtained within this new eroded mask. This procedure was repeated until a single pixel of the 3D platelet mask remained i.e. the center of the platelet had been reached.

### Whole genome sequencing (WGS)

WGS was performed on paired pDNA and cfDNA isolated simultaneously from the same peripheral blood samples of 15 patients with a recent diagnosis of gastrointestinal adenocarcinoma. Libraries were prepared from samples that were confirmed (using the Agilent Tapestation) to have both short DNA fragments of approximately 160 bp in addition to longer peaks of ~12’000 bp. Long platelet DNA was fragmented prior to library preparation. Briefly, 15 µl of DNA diluted in TE buffer (1mM Tris-HCl, pH 8.0, 0.1 mM EDTA) was added to a Covaris microtube-15 (Covaris, cat # 520145) and briefly centrifuged at 3000 x g for 1 minute. The microtube was loaded onto the Covaris ME220 Focused-ultrasonicator and the protocol run using the treatment conditions required for a 150 bp target peak. Fragment size was determined using the Agilent Tapestation 2200 system (Agilent Technologies) and the sonication was repeated if DNA fragments were > 150 bp.

DNA-Seq libraries were prepared using the NEBNext Ultra II DNA Sample Preparation Kit for Illumina (New England BioLabs, Ipswich, MA, USA) according to the manufacturer’s protocol. pDNA was size selected into 2 groups: short fragments (s-pDNA; under 600 bp) and long fragments (l-pDNA; over 600 bp). The short fragments were further cleaned to remove <100 bp fragments and large platelet fragments were fragmented via sonication as previously described. Following end repair and adapter ligation, adapter-ligated DNA fragments were amplified in enrichment PCR to generate final libraries. Cleaned up libraries were then multiplexed, and 1.5 pM libraries analyzed on an Illumina NextSeq 500 (300 cycle PE) at low-pass (0.1x) for all samples, and 10x for the cfDNA and s-pDNA, using four lanes in each sample with 4% PhiX to monitor sequencing performance. This amounted to 240 fastq files.

### Data pre-processing

Quality control of all fastq files was conducted by running FastQC (v0.11.8)(68). Reads were adaptor- and quality-trimmed using Trim Galore!(69) (v0.6.5) and aligned to the GATK Genome Reference Consortium Human Build 38 (GRCh38)(70) using bwa-mem (v0.7.17)(71). Reads were aligned to alternate contigs to represent common complex variation using bwa_postalt.js (https://github.com/lh3/bwa/blob/master/bwakit/README.md)(71). Resulting BAM files were merged, sorted and indexed with samtools (v1.13.0)(72).

GATK toolkit (v4.1.7.0) was used to mark optical and PCR duplicates, estimate library complexity, and calculate summary metrics on insert sizes(73). Mapping rate was evaluated with samtools flagstat and the number of reads mapped to each chromosome and the mitochondrial genome with idxstats. Repetitive elements in the ENCODE blacklist(74) were excluded using bedtools intersect (v2.30.0)(75).

### Insert size distribution

For all uniquely mapped, non-duplicated and properly paired read pairs, the frequency of insert size values was counted across all autosomes and chromosome X using samtools(72) and R. The distributions were plotted using ggplot2(76).

### Read depth around transcription start sites

Transcription start sites were defined by refTSS (v3.3)(77). We used the nearest refTSS entry to the 5’ coding sequence of all protein coding genes. Coverage per base in a 2 kilobase pair (kbp) region around each TSS was extracted with Rsamtools, counting only uniquely mapped, non-duplicate, correctly paired reads with insert sizes between 150 and 170 bp (“mono-nucleosome”) or between 280 and 350 bp (“di-nucleosome”). Peripheral blood mononuclear cell (PBMC) gene expression values were taken from Blueprint Epigenome experiment: EGAX00001327129 (http://dcc.blueprint-epigenome.eu/#/experiments/ERX1123729). Briefly, genes with posterior mean estimates of transcripts per million > 15 were defined as “highly expressed” in PBMC.

### Di-nucleosome peak difference quantification

A chi-square test of independence was performed in R with a simulated p-value to examine the relation between DNA type (cfDNA and s-pDNA) and the proportion of di-nucleosome fragments. We separated the fragments in (i) fragments below 250 bp representing mono-nucleosome fragments; and (ii) fragments above 250 bp representing di-nucleosome fragments. The proportion of di-nucleosome fragments was higher in s-pDNA than in cfDNA (*P* < 0.0005 in all samples).

### Chromosome coverage

Coverage was calculated using samtools bedcov, using a bed file of 10 kbp non-overlapping windows for all main chromosomes, but excluding reads with mapQ <1. Position with mappability (as defined by umap: https://bismap.hoffmanlab.org/) in the lowest 8 percentile were removed(78). The Coverage was then corrected for mappability and GC content bias using a linear regression model and visualized with karyoplotR(79).

### Gini index

After filtering for reads that are first in pair, uniquely mapped, mapped in proper pair, primary alignments, and non-duplicates, (samtools view parameters: -f 2 -F 3972 -q 1), we calculated the coverage with samtools depth. Evenness of coverage was evaluated by calculating the Gini index(80) on the short pDNA and cfDNA’s coverage, which was previously binned into adjacent 10 kbp bins.

### ichorCNA

Copy number alterations in the 10X and low-pass WGS were evaluated using ichorCNA (v0.2.0)(39) (github: https://github.com/broadinstitute/ichorCNA/releases/tag/v0.2.0).

### Tumor tissue DNA sequencing and analysis

DNA from resected tumor tissue and adjacent tumor-free colonic mucosa was extracted using the Monarch Genomic DNA Purification Kit (Monarch, cat # T3010S). For sequencing, gDNA was prepared as follows: 500 ng per sample at a concentration of 25 ng/µl. The tumor samples were sequenced at 75x and the normal samples at 30x by Novogene UK Ltd. The complete NextFlow pipeline for processing sequencing output to mutation calls is provided in the Zenodo code repository. Briefly, reads were trimmed using Trim Galore! (69) (v0.6.5), requiring >= 2bp adapter overlap and quality-trimming bases with Phred score below 20, and aligned against the complete hg38 genome, including alt alleles, using bwa-mem2 using default parameters. Mutations were called independently using Mutect2 (v4.1.7.0), Strelka (v2.9.10) and Octopus (v0.7.0). Mutations called by only one caller were excluded.

### Estimation of observed and expected tumor fragments

99 sets of random single-base mutations, each matching the unique mutation count identified in the tumor tissue of patient CRC_012, were generated by sampling from all SNVs detected in any WGS-sequenced colorectal cancer from TCGA. Any SNV overlapping a SNP (dbSNP v155) with > 0.5% allele frequency in 1000Genomes, HapMap, dbGaP_PopFreq, GnomAD_exomes, TOPMED or GnomAD were excluded, as well as variants identified as SNPs in the non-cancer control sample for patient CRC_012. Together with the original CRC_012 mutation set, this amounted to 100 mutation sets. For each set, reads covering the mutation positions were extracted, excluding improperly paired reads or reads with mapQ < 25. Detection rate was then calculated by dividing the count of reads containing the variant allele (min base quality: 10) by the total number of reads covering the mutation sites. Positions that exhibited an allele fraction of > 20% in plasma or platelet derived DNA were excluded as potential SNPs. The likelihood of observing a detection rate as high or higher than that of CRC_012 tissue mutations in that patient’s plasma and platelet-derived DNA was calculated using a normal distribution parameterized to the observed mean and standard deviation of detection rates for random mutation sets. Code and intermediate files are deposited in the publication’s Zenodo repository.

### TAPS sample preparation

Short (< 600 bp) and long (> 600 bp) platelet DNA was separated using 0.55X AMPure beads, followed by a 1.8X clean-up of the short platelet DNA. Genomic TAPS was carried out on long platelet DNA and cfDNA TAPS was carried out on short platelet DNA, as previously described(45, 81). Briefly, long platelet DNA was mixed with spike-ins of 0.1% CpG-methylated lambda phage DNA and 0.01% unmodified 2 kbp plasmid DNA and was sonicated and size-selected to 300-500 bp prior to library construction. Short platelet DNA was mixed with pre-fragmented spike-ins of the same ratio and used directly in library construction. DNA was ligated with KAPA Unique Dual-Indexed Adapter Kit using KAPA HyperPrep Kit according to the manufacturer’s protocol. The ligated DNA was subjected to mTet1-mediated oxidation and borane reduction. Converted DNA was then amplified with short primers ordered from IDT (FW-5’-AATGATACGGCGACCACCGAGATCTACAC-3’, RV-5’-CAAGCAGAAGACGGCATACGAGAT-3’) using KAPA HiFi HotStart Uracil+ ReadyMix PCR Kit for four cycles according to the manufacturer’s protocol. The PCR product was purified with AMPure XP beads. Finally, libraries were sequenced for 300 cycles in paired-end mode on the NextSeq 2000 with XLEAP-SBS chemistry P4 cartridge.

### TAPS data processing and analysis

Raw sequencing reads were trimmed from adapter sequences and low-quality bases using Trim Galore!(69) (v0.6.5) and assessed for quality with FastQC(68) (v0.11.9). Reads were aligned to the reference genome using BWA-MEM2(71) (v2.2.1) and duplicates were marked with Picard(73) (gatk, v4.4.0.0). BAM files were indexed and merged using samtools(72) (v1.16.1). TAPS conversion efficiency and methylation bias were estimated by analyzing sequencing spike-ins. Methylation levels were called using rastair (v0.8.1).

Sample cellular deconvolution was performed using WGBStools and UXM deconv as described in Loyfer et al. (2023)(46). A custom cellular atlas was built using the Loyfer et al. (2023) pat files for B cells, T cells, NK (natural killer) cells, blood granulocytes, monocytes, erythrocyte progenitors, hepatocytes and vascular endothelium, and an in-house megakaryocyte TAPS dataset. Lymphoid (B cells, T cells, and NK cells) and Myeloid (blood granulocytes, monocytes and erythrocyte progenitors) cells contributions were summed for visualization purposes. All code is available in the Zenodo repository.

## Code availability

Code used in these studies is available from Zenodo (82).

## Statistical analysis

Parametric tests, such as the paired Student’s *t* test, were used for comparisons within the same group when the data appeared normally distributed based on visual inspection. When data were paired but not normally distributed, nonparametric tests such as the Wilcoxon signed-rank test were applied. For comparisons between independent groups, the Mann-Whitney *U* test was used to assess statistical significance.

## Supplementary Material

Supplementary Material

## Data and materials availability

All data needed to evaluate the conclusions in the paper are present in the paper or the Supplementary Materials. Raw whole genome sequencing data has been deposited in the European Genome-phenome Archive (EGA) under accession number EGAS00001006854 (Study: EGA00001006854; Dataset EGAD00001009856; Data Access Committee: EGAC00001003034). Code is available from Zenodo (82).
