# Supplementary material for: Platelets sequester extracellular DNA, capturing tumor-derived and free fetal DNA
Source: Science. Author manuscript; Available in PMC 2025 Oct 10. (PMC7618233; doi:10.1126/science.adp3971)
Supplement: Supplementary Material [file EMS209376-supplement-Supplementary_Materials.pdf]

Supplementary Materials for

Platelets sequester extracellular DNA, capturing tumor-derived and free fetal DNA

Authors: Lauren Murphy[1], Jeanne Inchauspé[1,2], Giampiero Valenzano[3], Pamela Holland[4], Nikolaos Sousos[1,5], Hayley L Belnoue-Davis[6], Rong Li[1], Natalie J. Jooss[1], Camelia Benlabiod[1], Eleanor Murphy[1], Zohar Etzioni[2], Emelie Shepherd[2], Lucy Denly[2], Sujata Biswas[5], Lin Chen[2], Jennifer O'Sullivan[1], Michael P Rimmer[4], Abdullah O. Khan[1], Christina Simoglou Karali[1], Nadia Nasreddin[6], Ian S. Hitchcock[7], Milka Koupenova[8], Skirmantas Kriaucionis[2], Jim R. Hughes[1], Eric O'Neill[3], Manu Vatish[9], Paul Rees[10,11], Simon Leedham[6], Michael Desborough[1,5], Adam J. Mead[1,5], Benjamin Schuster-Böckler*[2], Christopher D. Gregory[4], Bethan Psaila*[1,2,5]

Correspondence to bethan.psaila@ndcls.ox.ac.uk. For computational enquiries: benjamin.schuster-boeckler@ludwig.ox.ac.uk

The PDF file includes:

Supplementary Text
Figs. S1 to S6
Tables S1 to S7
Supplementary Movies S1 to S6

Other Supplementary Materials for this manuscript include the following:

Movies S1 to S6
MDAR Reproducibility Checklist

SUPPLEMENTARY TEXT

$$r = \frac{\sum(I1_i - I1_{mean}) \times (I2_i - I2_{mean})}{\sqrt{\sum(I1_i - I1_{mean})^2 \times (I2_i - I2_{mean})^2}}$$

$$R = \frac{\sum I1_i \times I2_i}{\sqrt{\sum(I1_i)^2 \times (I2_i)^2}}$$

Equations used to calculate fluorescence correlation coefficients (Fig. 2H). To quantify signal co-localization between fluorescence channels, the Pearson's correlation coefficient (r) and Mander's overlap coefficient (R) were calculated for each platelet. In both equations $\mathrm{I1_i}$ and $\mathrm{I2_i}$ represent the fluorescence intensity at pixel *i* in channels 1 and 2, respectively. $I1_{\mathrm{mean}}$ and $I2_{\mathrm{mean}}$ denote the mean fluorescence intensity across all pixels in the respective channels.

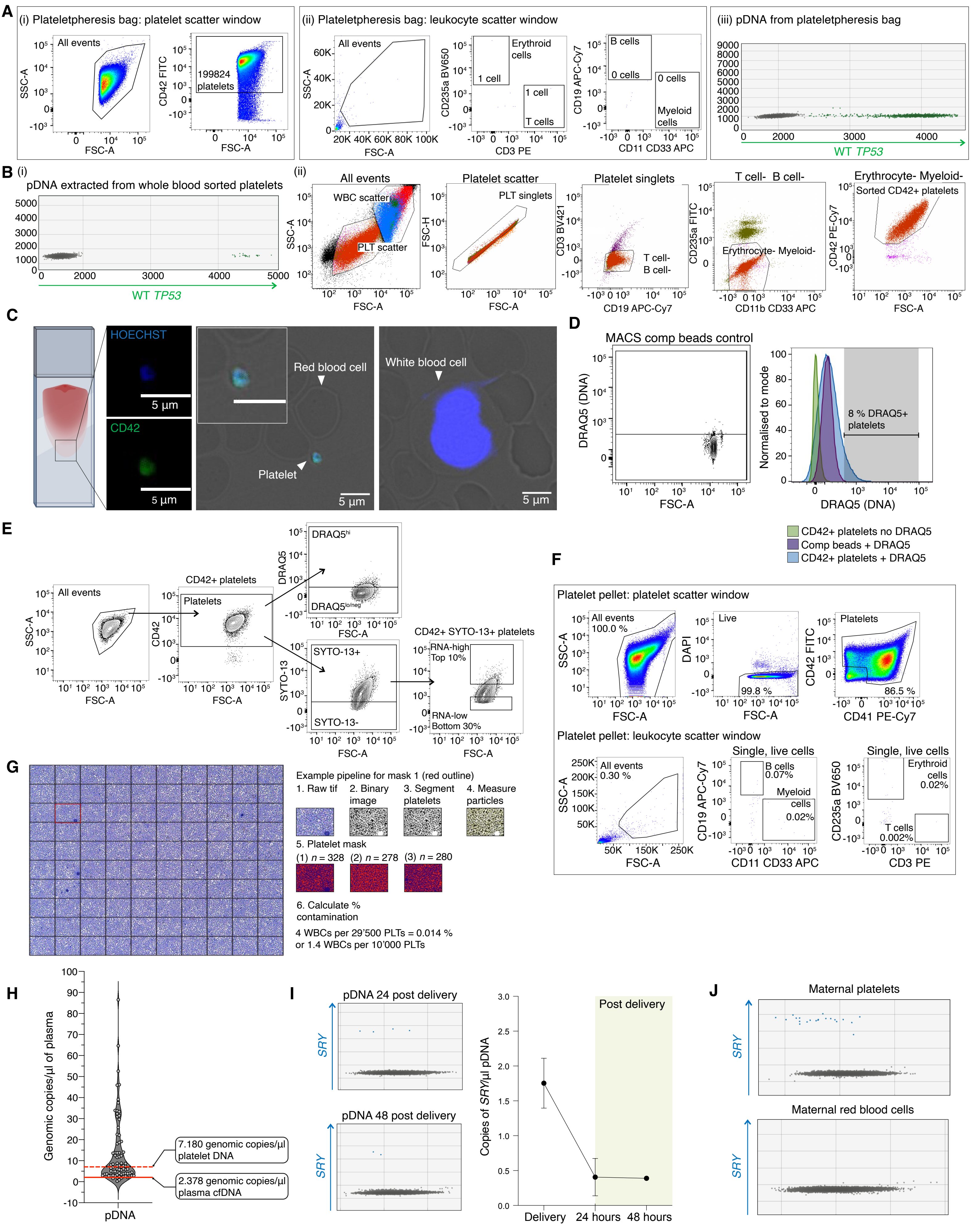
A
(i) Plateletpheresis bag: platelet scatter window
All events
199824 platelets
(ii) Plateletpheresis bag: leukocyte scatter window
Erythroid cells
1 cell
T cells
B cells
0 cells
Myeloid cells
(iii) pDNA from plateletpheresis bag
WT TP53
B
pDNA extracted from whole blood sorted platelets
WBC scatter
PLT scatter
PLT singlets
Platelet singlets
T cell- B cell-
Erythrocyte- Myeloid-
Sorted CD42+ platelets
C
HOECHST
CD42
Red blood cell
White blood cell
Platelet
5 µm
D
MACS comp beads control
8 % DRAQ5+ platelets
Normalised to mode
DRAQ5 (DNA)
E
CD42+ platelets
SYTO-13+
SYTO-13-
CD42+ SYTO-13+ platelets
RNA-high Top 10%
RNA-low Bottom 30%
CD42+ platelets no DRAQ5
Comp beads + DRAQ5
CD42+ platelets + DRAQ5
F
Platelet pellet: platelet scatter window
All events 100.0 %
Live 99.8 %
Platelets 86.5 %
Platelet pellet: leukocyte scatter window
All events 0.30 %
Single, live cells
B cells 0.07%
Myeloid cells 0.02%
Erythroid cells 0.02%
T cells 0.002%
G
Example pipeline for mask 1 (red outline)
1. Raw tif
2. Binary image
3. Segment platelets
4. Measure particles
5. Platelet mask
(1) n = 328 (2) n = 278 (3) n = 280
6. Calculate % contamination
4 WBCs per 29'500 PLTs = 0.014 %
or 1.4 WBCs per 10'000 PLTs
H
Genomic copies/µl of plasma
7.180 genomic copies/µl platelet DNA
2.378 genomic copies/µl plasma cfDNA
pDNA
I
pDNA 24 post delivery
pDNA 48 post delivery
SRY
Copies of SRY/µl pDNA
Post delivery
Delivery 24 hours 48 hours
J
Maternal platelets
Maternal red blood cells

Fig S1. Detection of DNA fragments and free fetal DNA in circulating blood platelets. **(A)** DNA was extracted from three healthy donor plateletpheresis concentrates. Flow cytometry confirms high platelet purity with minimal blood cell contamination. Plots show (i) platelet and (ii) leukocyte scatter with absolute cell counts. (iii) ddPCR detects wild-type (WT) *TP53* gene copies in platelets. **(B)** (i) ddPCR quantification of WT *TP53* gene copies from DNA extracted from 20.7 x 10^6 CD42+ platelets FACS-isolated from whole blood, following (ii) rigorous gating to exclude contaminating nucleated blood cell types (B cells, T cells and myeloid cells) and erythrocytes. *n* = 1 healthy donor. **(C)** Peripheral blood film from a healthy donor stained with CD42 AF488 (green) for platelets and Hoechst (blue) for DNA. Platelets show a Hoechst-positive signal. Red blood cells and white blood cells are also indicated. White box highlights a magnified region, scale bars represent 5 µm. Representative image acquired using a Leica SPE. **(D)** Representative flow cytometric analysis of MACS compensation beads stained with DRAQ5 to control for DRAQ5 non-specific binding (*n* = 1 healthy donor). Right, thresholding to identify strongly DRAQ5+ platelets (*n* = 20 healthy donors, blue; concatenated into a single histogram), defined by fluorescence exceeding that of compensation beads (*n* = 1 donor, purple). **(E)** Flow cytometric gating strategy for defining newly produced (RNA-high) and older (RNA-low) platelets. 'Young' platelets are identified as being within the top 10% of SYTO-13 staining, while 'old' are within the bottom 30%. *n* = 10 healthy donors, concatenated data shown. **(F)** Flow cytometric analysis of a representative platelet pellet from a healthy donor (*n* = 1 donor) confirming high platelet yield with minimal leukocyte and red blood cell contamination. Platelet (top) and leukocyte (bottom) scatter windows are shown, using thresholds of 1000 for platelets and 5000 for leukocytes and red blood cells. **(G)** Cytospin with Wright-Giemsa staining and leukocyte contamination quantification in a representative colorectal patient platelet pellet (*n* = 1 patient). Imaged at 40X using an Olympus BX51 fluorescent microscope. **(H)** Violin plot showing DNA yield from platelet pellets and platelet-depleted plasma, measured as genomic equivalents per µl of plasma. *n* = 76 donors, including non-cancer controls, patients with pre-malignant serrated polyps and gastrointestinal carcinoma. Each data point represents one patient. Dashed line indicates the median DNA yield from platelet pellets (7.180 genomic copies/µl of platelet-rich plasma), while solid line marks the median yield of DNA from paired platelet-depleted plasma (cfDNA, 2.378 genomic copies/µl of plasma). **(I)** Top, representative ddPCR detection of the Y-chromosome gene *SRY* in maternal platelets 24 and 48 hours post-cesarean delivery of a male infant. Bottom, mean *SRY* copies ± SEM, in maternal platelets at delivery (*n* = 9 donors), 24 hours (*n* = 5) and 48 hours post-delivery (*n* = 2). **(J)** Representative ddPCR showing detection of the Y-chromosome gene *SRY* in maternal platelets, but not red blood cells sampled from mothers of male offspring prior to delivery. ddPCR, droplet digital PCR; FACS, fluorescence activated cell sorting; pDNA, platelet DNA; *SRY*, sex-determining region Y; WT, wild type.

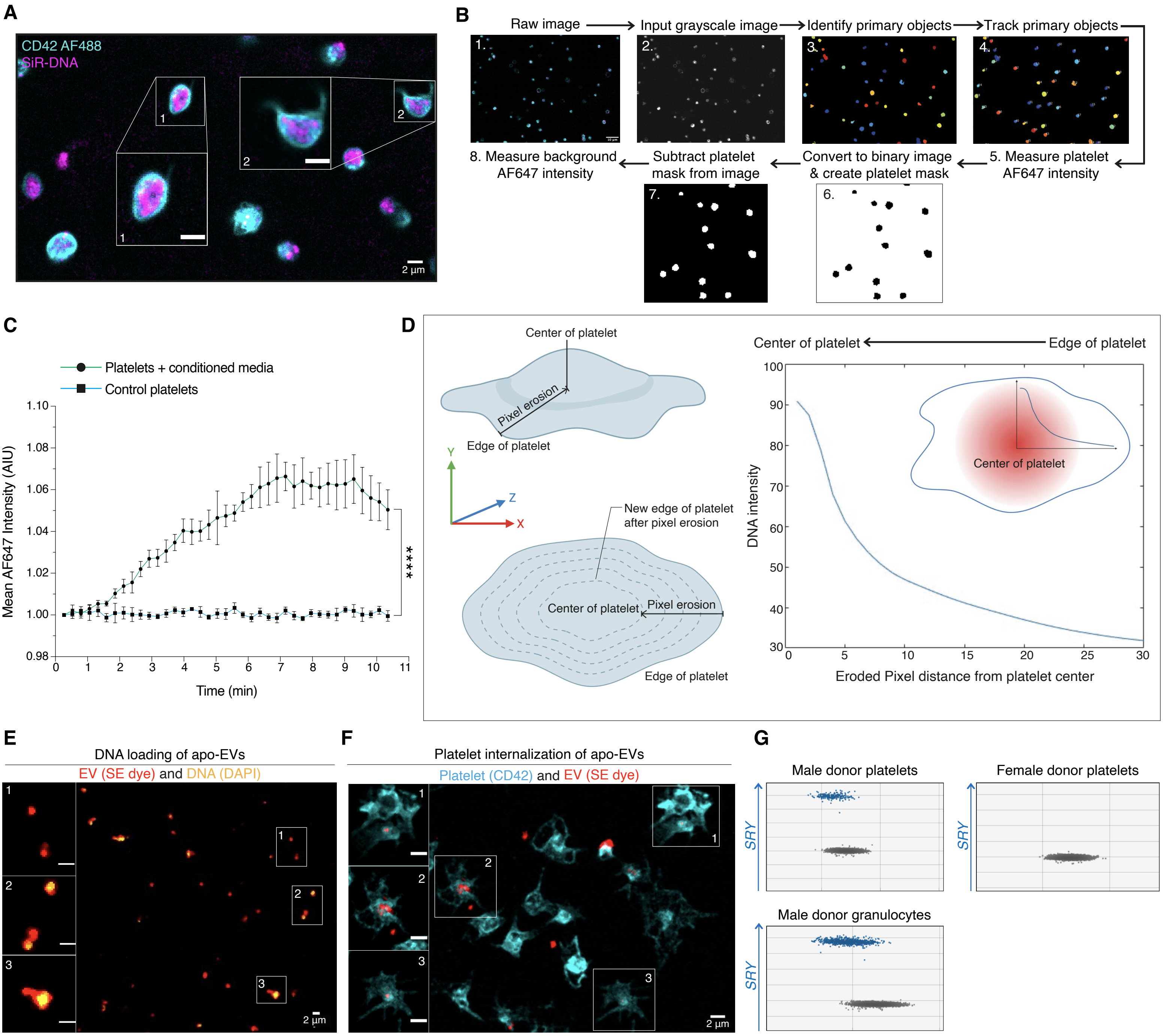
A
CD42 AF488
SiR-DNA
2 µm
B
Raw image
Input grayscale image
Identify primary objects
Track primary objects
5. Measure platelet AF647 intensity
Convert to binary image & create platelet mask
Subtract platelet mask from image
8. Measure background AF647 intensity
C
Platelets + conditioned media
Control platelets
Mean AF647 Intensity (AIU)
Time (min)
****
D
Center of platelet
Pixel erosion
Edge of platelet
New edge of platelet after pixel erosion
Center of platelet ← Edge of platelet
DNA intensity
Eroded Pixel distance from platelet center
E
DNA loading of apo-EVs
EV (SE dye) and DNA (DAPI)
2 µm
F
Platelet internalization of apo-EVs
Platelet (CD42) and EV (SE dye)
2 µm
G
Male donor platelets
Female donor platelets
Male donor granulocytes
SRY

Fig S2. Platelet sequestration of DNA released by nucleated cells. (A) COLO205 colorectal carcinoma cells were labeled with SiR-DNA (magenta), washed twice and co-incubated with CD42 AF488 labeled healthy donor platelets (cyan). White box highlights COLO205-derived DNA in platelets. Images acquired on a Zeiss Spinning Disk confocal at 63X magnification, representative platelets shown. **(B)** CellProfiler method to quantify fluorescently labeled DNA uptake by platelets over time: (1-2) convert raw images to grayscale, (3) segment platelets, (4) track individual platelets over 10 minutes, (5) calculate average platelet DNA (AF647) signal per frame, and (6-8) subtract background intensity. **(C)** Time-course quantification of platelet DNA fluorescence intensity presented as mean ± SD. ****$P < 0.0001$; as determined by a Wilcoxon signed-rank test. Tracking of five randomly selected platelets incubated with control media (n = 5 platelets) or media conditioned by NUCLEAR-ID-labeled COLO205 cells (n = 5 platelets). AF647 signal normalized to frame 1. **(D)** Left, schematic outlining the method used to measure DRAQ5 pixel intensity in CD62P labeled, healthy donor platelets spread on collagen. CD62P was used to generate a 3D platelet mask which was subsequently eroded pixel-by-pixel towards the center of the platelet. Intensities along the X, Y, and Z planes were calculated. Right, pixel intensity (y axis) was plotted against distance from platelet center (x axis). DNA (DRAQ5) signal is strongest at the center of the platelet. Data collected from n = 1 healthy donor platelet. **(E)** Representative BL2 cell derived apo-EVs labeled with an amine-reactive membrane dye (CF658, red), stain positive for the DNA stain, DAPI (yellow). Imaged using a ZEISS LSM900, 63X magnification, scale bars represent 2 μm. **(F)** Following incubation with labeled EVs, platelets were stained with CD42 AF488 (cyan). EVs (red) were visualized in platelets, confirming platelet uptake of apo-EVs. White boxes show magnified regions, scale bars represent 2 μm. **(G)** Representative ddPCR detection of the Y-chromosome gene *SRY* in male donor platelets and granulocytes but not in female donor platelets. AIU, Arbitrary intensity units; BL2, Burkitt's lymphoma cells; ddPCR, droplet digital PCR; SE, succinimidyl ester; *SRY*, sex-determining region Y.

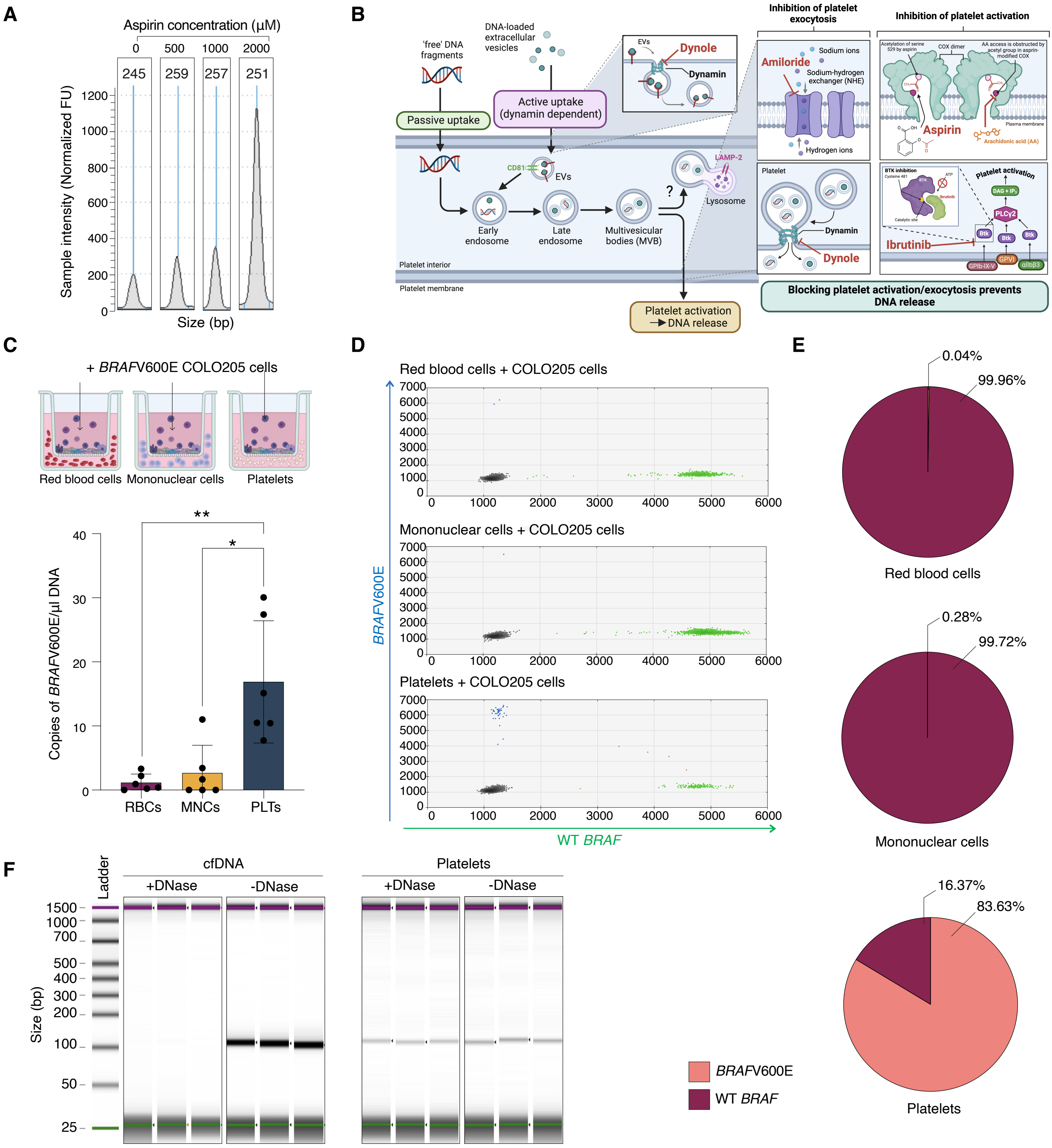

A
Aspirin concentration (µM)
0
500
1000
2000
245
259
257
251
Sample intensity (Normalized FU)
Size (bp)
B
'free' DNA fragments
DNA-loaded extracellular vesicles
Passive uptake
Active uptake (dynamin dependent)
EVs
Dynole
Dynamin
CD81
Early endosome
Late endosome
Multivesicular bodies (MVB)
LAMP-2
Lysosome
Platelet interior
Platelet membrane
Platelet activation → DNA release
Inhibition of platelet exocytosis
Amiloride
Sodium ions
Sodium-hydrogen exchanger (NHE)
Hydrogen ions
Platelet
Inhibition of platelet activation
Acetylation of serine 529 by aspirin
COX dimer
AA access is obstructed by acetyl group in aspirin-modified COX
Aspirin
Plasma membrane
Arachidonic acid (AA)
BTK inhibition
Platelet activation
DAG + IP3
PLCγ2
Ibrutinib
Btk
GPIb-IX-V
GPVI
αIIbβ3
Blocking platelet activation/exocytosis prevents DNA release
C
+ BRAFV600E COLO205 cells
Red blood cells
Mononuclear cells
Platelets
Copies of BRAFV600E/µl DNA
**
*
RBCs
MNCs
PLTs
D
Red blood cells + COLO205 cells
Mononuclear cells + COLO205 cells
Platelets + COLO205 cells
BRAFV600E
WT BRAF
E
0.04%
99.96%
Red blood cells
0.28%
99.72%
Mononuclear cells
16.37%
83.63%
Platelets
BRAFV600E
WT BRAF
F
Ladder
cfDNA
Platelets
+DNase
-DNase
Size (bp)
1500
1000
700
500
400
300
200
100
50
25

Fig S3. Mechanisms of DNA trafficking by platelets and detection of cancer cell-derived DNA in platelets. (A) Representative electrophoresis showing recovery of 260 bp synthetic DNA fragments from platelets treated with increasing concentrations of aspirin (0, 500, 1000 and 2000 µM), n = 3 independent experiments. **(B)** Schematic illustrating DNA trafficking mechanisms. Uptake of synthetic, non-membrane-encapsulated DNA was unaffected by inhibitors, while DNA-loaded vesicle uptake was reduced by dynamin inhibition (Dynole), indicating distinct uptake pathways. Inhibitors of platelet activation (aspirin, ibrutinib) and exocytosis (Dynole, amiloride) increased DNA yield from platelet pellets by blocking DNA release. Inhibitor mechanisms are shown. **(C)** Top, schematic showing co-culture of COLO205 cells with red blood cells, mononuclear cells and platelets. Bottom, quantification of *BRAF*V600E in DNA extracted from each cell type following co-culture with COLO205 cells. Data points show mean ± SD, each point represents 1 co-culture experiment, n = 6 replicate wells from three independent experiments, *$P < 0.05$; **$P < 0.01$; as determined by a Mann-Whitney U test. **(D)** Representative ddPCR for *BRAF*V600E in DNA isolated from red blood cells, mononuclear cells and platelets following incubation in COLO205 cell conditioned media. **(E)** Percentage detection of *BRAF* wild-type and mutant alleles in red blood cells, mononuclear cells and platelets. Data collected from n = 6 replicate wells from three independent incubation experiments. **(F)** Representative electrophoresis showing recovery of synthetic, 128 bp DNA fragments from healthy donor derived platelets after treatment with or without DNase, n = 3 independent experiments. MNCs, mononuclear cells; PLTs, platelets; RBCs, red blood cells; WT, wild type.

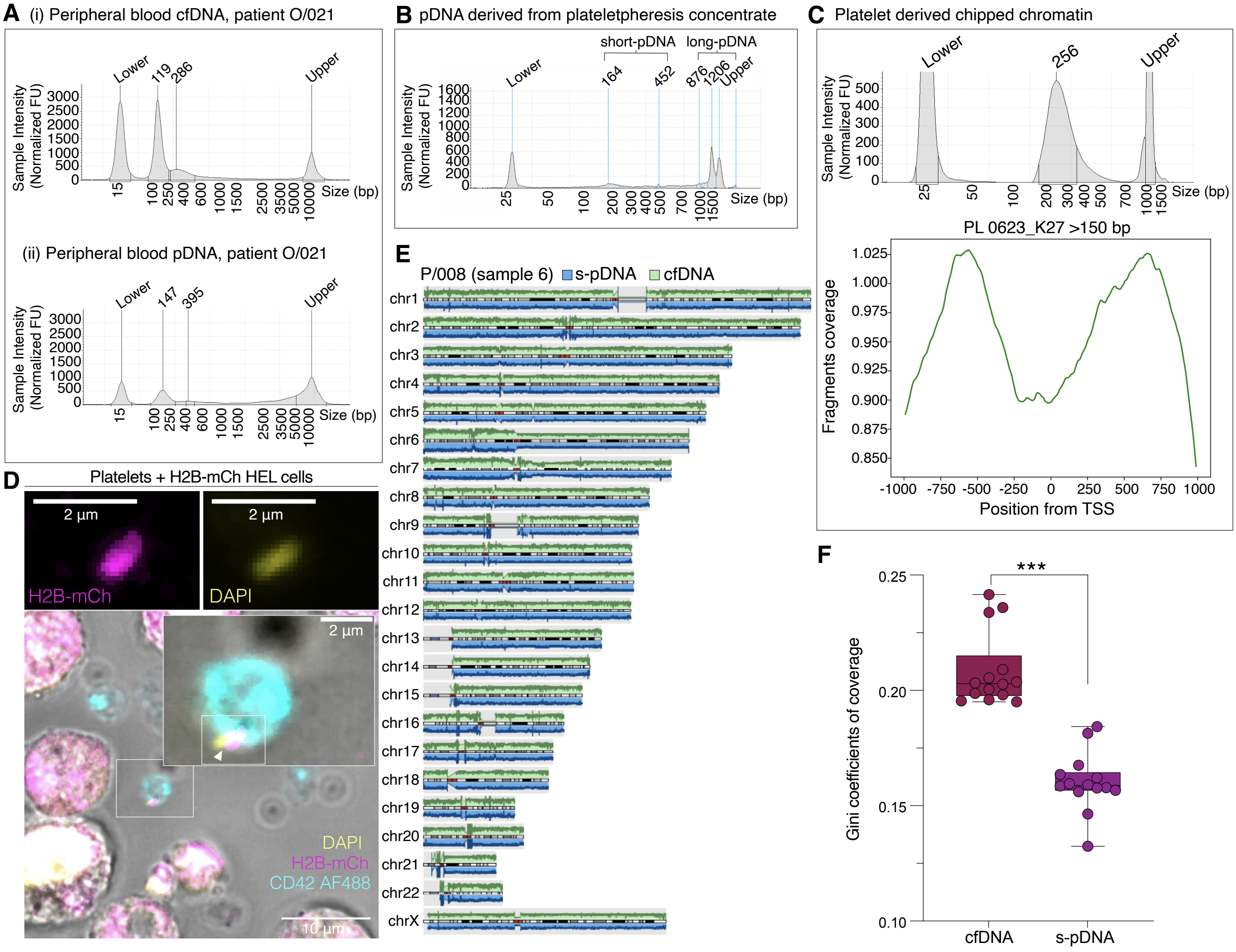
A (i) Peripheral blood cfDNA, patient O/021
Sample Intensity (Normalized FU)
Lower
119
286
Upper
Size (bp)
(ii) Peripheral blood pDNA, patient O/021
Lower
147
395
Upper
B pDNA derived from plateletpheresis concentrate
short-pDNA
long-pDNA
Lower
164
452
876
1206
Upper
C Platelet derived chipped chromatin
Lower
256
Upper
PL 0623_K27 >150 bp
Fragments coverage
Position from TSS
D Platelets + H2B-mCh HEL cells
2 µm
H2B-mCh
DAPI
CD42 AF488
10 µm
E P/008 (sample 6) s-pDNA cfDNA
chr1
chr2
chr3
chr4
chr5
chr6
chr7
chr8
chr9
chr10
chr11
chr12
chr13
chr14
chr15
chr16
chr17
chr18
chr19
chr20
chr21
chr22
chrX
F
***
Gini coefficients of coverage
cfDNA
s-pDNA

Fig S4. Platelets capture fragments of DNA that map across the human genome, including DNA fragments from cancer cells. (A) Representative electrophoresis of DNA extracted from (i) plasma and (ii) peripheral blood platelets, showing peaks at ~150 bp and nucleosomal footprints. **(B)** Representative electrophoresis of platelet DNA extracted from one of three plateletpheresis samples showing a peak at ~160 bp as well as larger DNA fragments, *n* = 3 plateletpheresis donors. **(C)** Top, representative electrophoresis of a H3K27ac ChIPmentation DNA library from platelet chromatin. Bottom, coverage of the H3K27ac ChIP-seq reads at the TSS, for nucleosomal reads (bioinformatically size selected to >= 150 bp). The typical low coverage at the TSS, flanked by regions of higher coverage, suggests open chromatin with surrounding nucleosomes marked by H3K27ac at the TSS. This result suggests DNA in platelets is in chromatin form and contains promoter signal. The signal was smoothed with a Savitzky-Golay filter and repeated in *n* = 2 plateletpheresis donors. **(D)** Representative image of healthy donor platelets (CD42 AF488, cyan) incubated with H2B-mCh HEL cells genetically engineered to express mCherry-tagged histones (magenta). Arrow shows platelet sequestration of tumor cell-derived histones (magenta) and DNA (DAPI, yellow). White box shows magnified region, scale bars represent 2 µm, imaged using a Zeiss Spinning Disk Confocal with a 63X oil immersion objective. **(E)** Chromosome mapping of short platelet DNA and paired cfDNA for a representative patient with pancreatic adenocarcinoma (P/008, sample 6, *n* = 1). **(F)** s-pDNA fragments have more even chromosome coverage, as indicated by a significantly lower Gini index compared to cfDNA as calculated by a Wilcoxon signed-rank test, ****P* < 0.001; *n* = 14 patients with gastrointestinal carcinoma. In the box plot, the center line represents the median, whiskers indicate the minimum and maximum values, and dots represent individual patients. cfDNA, cell free DNA; pDNA, platelet DNA; s-pDNA, short platelet DNA; TSS, transcription start sites.

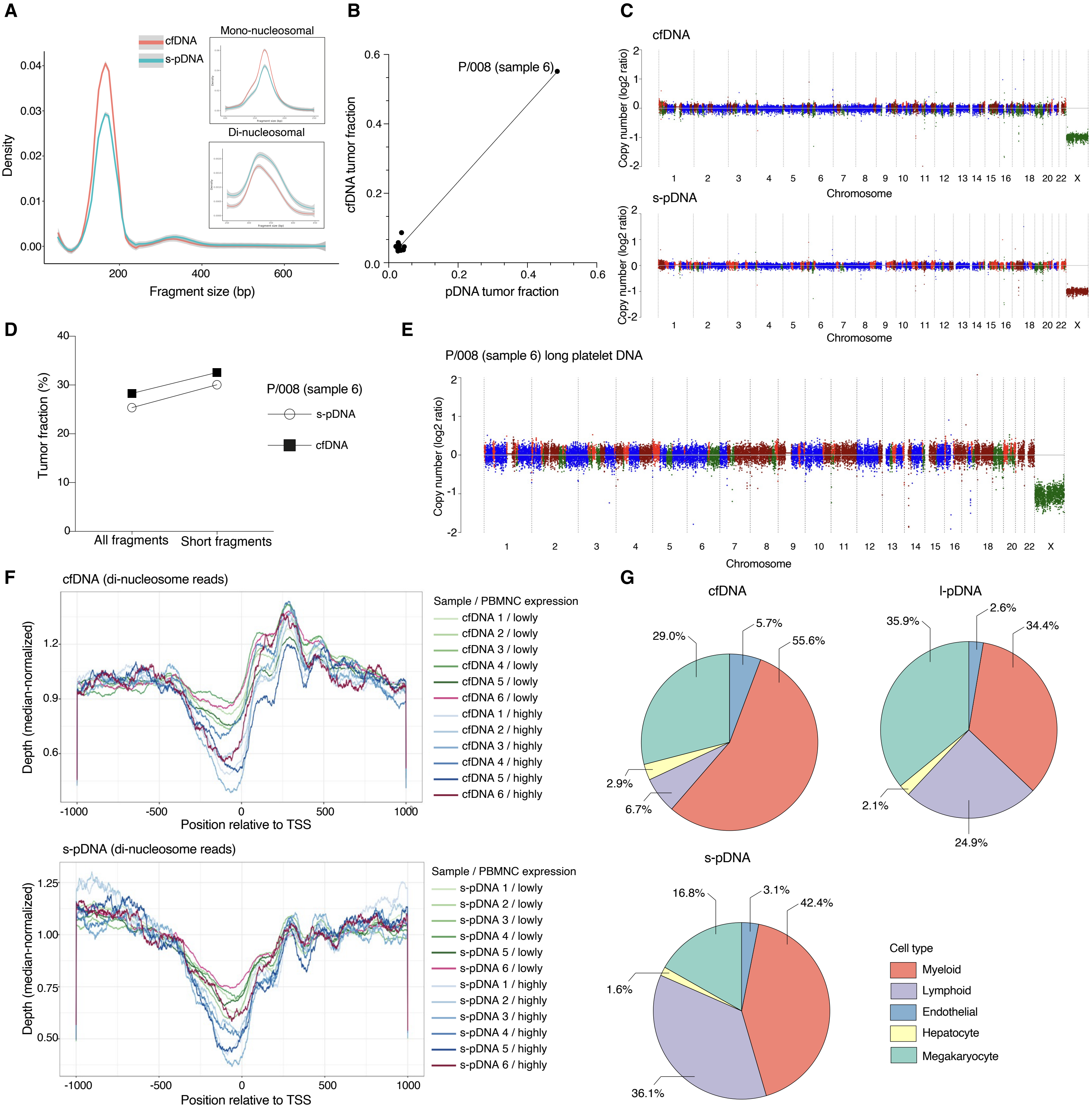

A
cfDNA
s-pDNA
Mono-nucleosomal
Di-nucleosomal
Density
0.04
0.03
0.02
0.01
0.00
200
400
600
Fragment size (bp)
B
P/008 (sample 6)
cfDNA tumor fraction
pDNA tumor fraction
0.0
0.2
0.4
0.6
C
cfDNA
s-pDNA
Copy number (log2 ratio)
1 2 3 4 5 6 7 8 9 10 11 12 13 14 15 16 18 20 22 X
Chromosome
D
Tumor fraction (%)
40
30
20
10
0
All fragments
Short fragments
P/008 (sample 6)
s-pDNA
cfDNA
E
P/008 (sample 6) long platelet DNA
Copy number (log2 ratio)
Chromosome
F
cfDNA (di-nucleosome reads)
Depth (median-normalized)
Position relative to TSS
Sample / PBMNC expression
cfDNA 1 / lowly
cfDNA 2 / lowly
cfDNA 3 / lowly
cfDNA 4 / lowly
cfDNA 5 / lowly
cfDNA 6 / lowly
cfDNA 1 / highly
cfDNA 2 / highly
cfDNA 3 / highly
cfDNA 4 / highly
cfDNA 5 / highly
cfDNA 6 / highly
s-pDNA (di-nucleosome reads)
s-pDNA 1 / lowly
s-pDNA 2 / lowly
s-pDNA 3 / lowly
s-pDNA 4 / lowly
s-pDNA 5 / lowly
s-pDNA 6 / lowly
s-pDNA 1 / highly
s-pDNA 2 / highly
s-pDNA 3 / highly
s-pDNA 4 / highly
s-pDNA 5 / highly
s-pDNA 6 / highly
-1000
-500
0
500
1000
G
cfDNA
29.0%
5.7%
55.6%
2.9%
6.7%
l-pDNA
35.9%
2.6%
34.4%
2.1%
24.9%
s-pDNA
16.8%
3.1%
42.4%
1.6%
36.1%
Cell type
Myeloid
Lymphoid
Endothelial
Hepatocyte
Megakaryocyte

Fig S5. Platelet DNA analysis reveals copy number variations and cell-of-origin. (A) Plot showing significantly higher frequency of di-nucleosomal fragments (fragments > 250 bp) in s-pDNA than cfDNA ($P < 0.0005$ for all samples as determined by a chi-square test of independence). $n = 14$ patients with gastrointestinal carcinoma, insets show zoomed in mono- and di-nucleosomal peaks. The standard error of the mean is shown in gray. **(B)** CNA-based estimates of tumor fraction in cfDNA and pDNA are highly correlated, $n = 14$ patients with gastrointestinal carcinoma, each dot represents one patient. **(C)** Deviation from median coverage in 100 kbp windows across chromosomes for cfDNA and s-pDNA for a patient with colorectal carcinoma who had a curative right hemicolectomy prior to blood sampling ($n = 1$ patient). **(D)** Estimated tumor fraction increases after computationally selecting DNA fragments smaller than 150 bp for both s-pDNA and cfDNA for P/008 (sample 6, $n = 1$). **(E)** Copy number alterations were not observed in long platelet DNA for the same patient (P/008, sample 6, $n = 1$). **(F)** Read depth distribution around transcription start sites of genes highly (TPM >15, blue) or lowly (TPM <15, green) expressed in peripheral blood mononuclear cells for cfDNA (top) and s-pDNA (bottom) di-nucleosome-length reads. $n = 6$ donors with gastrointestinal carcinoma, depth per position per sample was normalized to the median read depth across all genes in that expression category. **(G)** Cell type deconvolution of low-input whole genome DNA methylation data to compare cell-of-origin of cfDNA ($n = 10$ healthy donors), long platelet DNA ($n = 6$ healthy donors) and short platelet DNA ($n = 6$ healthy donors). Long and short DNA were from matched donors. 'Lymphoid' contributions include B, T, and NK cells, while 'Myeloid' contributions include granulocytes, monocytes, and erythrocytes. CNA, copy number analysis; kbp, kilobase pair; NK, natural killer; PBMNC, peripheral blood mononuclear cell; TPM, transcripts per million; TSS, transcriptional start site.

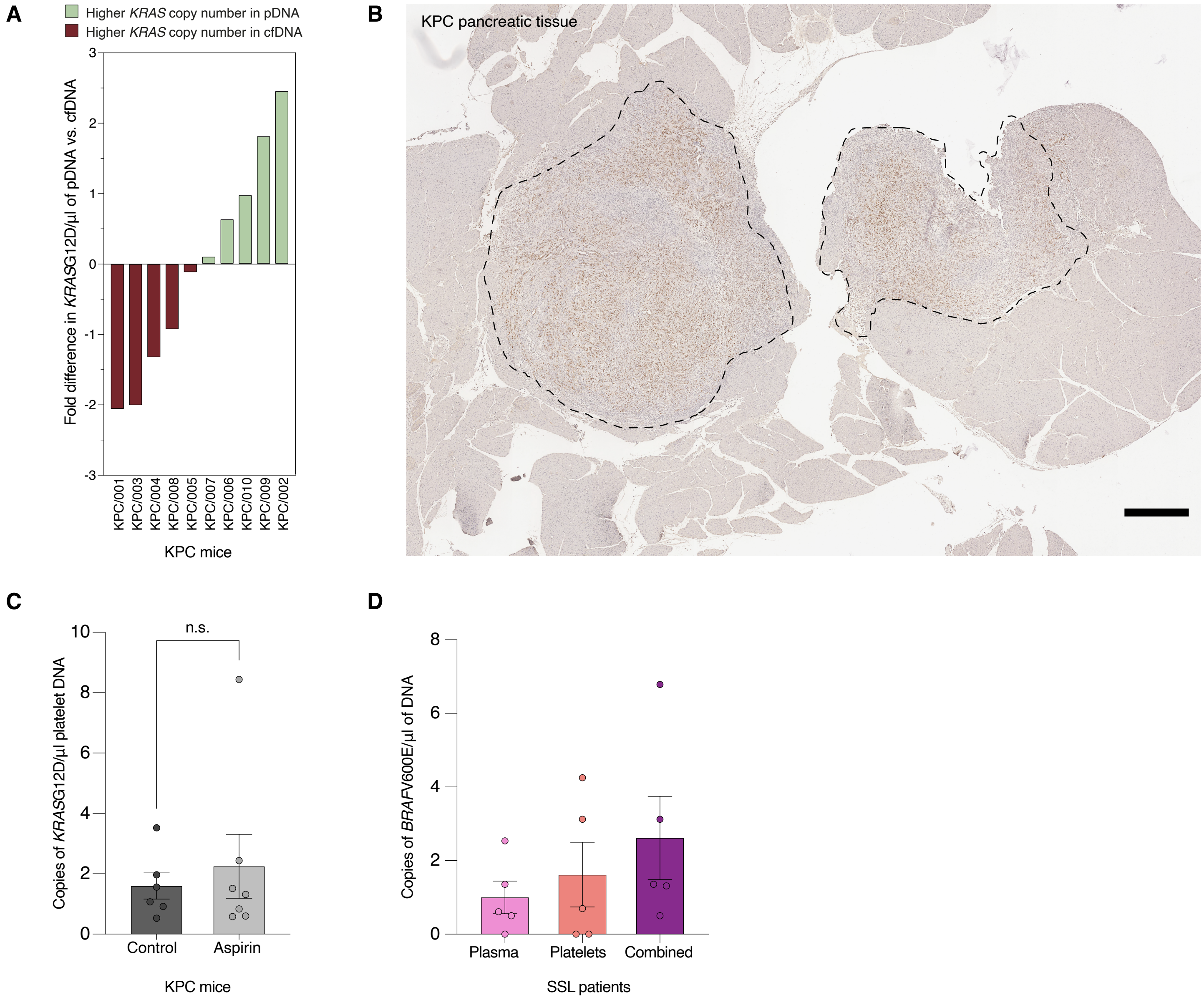

A
Higher KRAS copy number in pDNA
Higher KRAS copy number in cfDNA
Fold difference in KRASG12D/µl of pDNA vs. cfDNA
3
2
1
0
-1
-2
-3
KPC/001
KPC/003
KPC/004
KPC/008
KPC/005
KPC/007
KPC/006
KPC/010
KPC/009
KPC/002
KPC mice
B
KPC pancreatic tissue
C
Copies of KRASG12D/µl platelet DNA
10
8
6
4
2
0
n.s.
Control
Aspirin
KPC mice
D
Copies of BRAFV600E/µl of DNA
8
6
4
2
0
Plasma
Platelets
Combined
SSL patients

Fig S6. DNA abundance in platelets and plasma of mice with pancreatic cancer. (A) Waterfall plot showing fold difference in copies of *KRAS*G12D detected using ddPCR per µl of DNA extracted from the plasma (cfDNA) and platelets (pDNA) of KPC mice, two weeks after tumor cell implantation, *n* = 10 mice, each bar represents one mouse. **(B)** Representative image showing p53 immunohistochemical staining of KPC pancreatic tissue, two weeks post engraftment. Outlined area highlights regions of contained p53 expression surrounded by healthy p53neg tissue. Scale bar represents 500 µm. **(C)** Copies of *KRAS*G12D detected in DNA extracted from the platelets of KPC mice, normalized to cfDNA copies per mouse, to account for aspirin's effect on tumor growth. Mice were treated with aspirin (10 mg/kg) or control for two weeks, starting four days after tumor cell implantation. >1 indicates more *KRAS*G12D copies in platelets compared to paired cfDNA. Individual points represent one mouse with mean ± SEM, *n* = 6 control mice and *n* = 7 aspirin treated mice, n.s., not significant as determined by paired *t* test. **(D)** ddPCR quantification of mutant *BRAF*V600E copies per µl of DNA from platelet-poor plasma and platelets in five patients with SSLs, where mutant *BRAF* was detected in either patient cfDNA or pDNA (*n* = 5 SSL patients) . The combined cfDNA and platelet signal is also shown. Each datapoint represents one patient, mean ± SEM. ddPCR, droplet digital PCR; KPC, LSL-*Kras*G12D, LSL-*Trp53*R172H, *Pdx1*Cre; SSL, sessile serrated lesions.

SUPPLEMENTARY TABLES

Patient ID	Blood collection tube	Age (years)	Sex	Platelet count ($x10^9$/L)
NC/001	Streck	35	M	N/A
NC/003	Streck	63	M	303
NC/004	Streck	66	F	211
NC/005	Streck	65	M	330
NC/006	Streck	53	F	282
NC/007	Streck	28	F	200
NC/008	Streck	42	M	343
NC/009	Streck	61	M	255
NC/010	Streck	41	F	398
NC/011	Streck	69	F	216
NC/012	Streck	28	M	270
NC/013	Streck	47	F	398
NC/015	Streck	72	M	N/A
NC/018	Streck	81	M	137

Table S1: Clinical information for the control cohort (*n* = 14, including patients with ulcerative colitis, Crohn's disease and individuals on the bowel cancer screening program).

F, female; M, male; N/A, not available; NC, non-cancer control

Patient ID	Blood collection tube	Age (years)	Sex	Platelet count ($x10^9/L$)	Diagnosis
SSL/001*	Streck	43	F	260	Confirmed SSL and on surveillance
SSL/002*	Streck	38	F	N/A	Confirmed SSL and on surveillance
SSL/003*	Streck	37	M	274	Confirmed SSL and on surveillance
SSL/004	Streck	73	M	205	Confirmed SSL and on surveillance
SSL/005	Streck	41	M	92	Confirmed SSL and on surveillance for SSLs as well as previous surgery for CRC
SSL/006*	Streck	52	F	351	Confirmed SSL and on surveillance
SSL/007	Streck	75	M	270	Confirmed SSL and on surveillance
SSL/007.2	Streck	76	M	N/A	Confirmed SSL and on surveillance
SSL/008	Streck	38	M	N/A	Surveillance after previous polyps, family history of CRC and SSL
SSL/009*	Streck	35	F	N/A	Confirmed SSL and on surveillance
SSL/010	Streck	43	F	188	Confirmed SSL and on surveillance
SSL/012	Streck	44	F	317	Not confirmed SSL but on surveillance
SSL/013	Streck	75	F	348	Confirmed SSL and on surveillance
SSL/018	Streck	41	F	276	Confirmed SSL and on surveillance
SSL/019	Streck	48	F	208	Confirmed SSL and on surveillance
SSL/022	Streck	43	F	N/A	Confirmed SSL and on surveillance
SSL/024	Streck	40	F	246	Confirmed SSL and on surveillance
SSL/025	Streck	43	F	N/A	Confirmed SSL and on surveillance
SSL/026	Streck	43	M	N/A	Confirmed SSL and on surveillance
SSL/027	Streck	70	F	N/A	Confirmed SSL and on surveillance
SSL/028	Streck	73	F	N/A	Confirmed SSL and on surveillance
SSL/029	Streck	43	M	N/A	Confirmed SSL and on surveillance
SSL/030	Streck	42	M	N/A	Confirmed SSL and on surveillance
SSL/30.2	Streck	43	M	215	Confirmed SSL and on surveillance
SSL/031	Streck	62	F	229	Confirmed SSL and on surveillance
SSL/032	Streck	43	M	N/A	Confirmed SSL and on surveillance
SSL/033	Streck	25	F	N/A	Familial adenomatous polyposis and on surveillance
SSL/034	Streck	41	M	226	Confirmed SSL and on surveillance
SSL/035	Streck	40	M	251	Confirmed SSL and on surveillance
SSL/037	Streck	44	F	N/A	Confirmed SSL and on surveillance
SSL/038	Streck	45	M	N/A	Confirmed SSL and on surveillance
SSL/039	Streck	41	F	N/A	Confirmed SSL and on surveillance
SSL/040	Streck	56	F	278	Confirmed SSL and on surveillance
SSL/041	Streck	59	M	251	Confirmed SSL and on surveillance

Table S2: Clinical information for patients with pre-malignant sessile serrated lesions (*n* = 34).

* SSL patients where *BRAF*V600E was detected in either cfDNA or pDNA
CRC, colorectal cancer; F, female; M, male; N/A, not available; SSL, sessile serrated lesions

Patient ID	Blood collection tube	Cancer type	Platelet count ($x10^9/L$)	Tumor information (location, size)	TNM staging	Additional pathologic features	Identified mutations	Therapy
C/024	Streck	Colorectal	332	Ascending colon, caecum with intramural lymphatic invasion. Tumor size: 5.5 cm	T4a N1a Mx	N/A	None identified	N/A
C/025	Streck	Colorectal	248	Sigmoid cancer with liver metastasis	T3 N2 M1	N/A	*TP53* c.578A>G p.(His193Arg)	4 cycles of chemotherapy (oxaliplatin and capecitabine)
C/027	EDTA	Colorectal	235	Transverse colon	T3 N0 M0	N/A	*TP53* c.733G>A p.(Gly2455Ser)	N/A
C/028	EDTA	Colorectal	323	Ascending colon	T2 N1 M0	N/A	*TP53* c.659A>G p.(Tyr220Cys)	N/A
C/029	EDTA	Colorectal	183	Rectosigmoid tumor	T4a N1 Mx	pT4a pN1b Ly1 V1 R0	*TP53* c.742C>T p.(Arg248Trp). *KRAS* c.37G>T p.(Gly13Cys)	N/A
C/031	Streck	Colorectal	317	Ascending colon. Tumor size: 22 mm	T2 N0 Mx	pT2 pN0 Ly0 V0 R0	*KRAS*: c.34G>T p.(Gly12Cys). *PIK3CA*: c.1258T>C p.(Cys420Arg).*TP53*: c.691A>C p.(Thr231Pro)	N/A
O/030	Streck	Esophageal	193	Proximal/mid oesophagus. Tumor size: 3.5 cm	T2 N1 M0	R1 pT3 L1 V0 Pn0 N1 [2/19] M0	None identified	2 cycles of neoadjuvant chemotherapy (cisplatin and fluorouracil) prior to surgery
O/019	EDTA	Esophageal	225	Oesophagogastric junction. Tumor size: 63 mm (30 mm above + 33 mm below)	T1a N0 Mx	R0 ypT1a L0 V0 Pn0 N0	None identified	4 cycles of neoadjuvant FLOT chemotherapy, 2 months prior to blood sampling

Table S3: Clinical information for patients with gastrointestinal carcinoma (*n* = 15).

FLOT, Combination of chemotherapy drugs: docetaxel, oxaliplatin, 5-fluorouracil and leucovorin; GOJ, gastro-esophageal junction

Patient ID	Blood collection tube	Cancer type	Platelet count ($x10^9/L$)	Tumor information (location, size)	TNM staging	Additional pathologic features	Identified mutations	Therapy
O/023	EDTA	Esophageal	261	Gastric cardia just below GOJ. Tumor size: 45 mm	T4a N2 M1	R1 ypT4a L1 Pn1 N2 (4/35) M1	None identified	4 cycles of neoadjuvant FLOT chemotherapy 1 month prior to blood sampling
O/024	Streck	Esophageal	189	Tumor at GOJ, size: 30 mm	T3 N0 M0	R0 ypT3 L0 V0 Pn0 N0 (0/22) M0	None identified	Post neoadjuvant FLOT chemotherapy and radiotherapy after blood sampling
O/025	Streck	Esophageal	N/A	N/A	N/A		N/A	N/A
O/026	Streck	Esophageal	270	Tumor at lower 3rd distal oesophagus and GOJ. Tumor size: 30 mm x 45 mm	T3 N2 M0	R0 ypT3 L1 V0 Pn1 N2	None identified	4 cycles of neoadjuvant FLOT and 5FU, 3 months prior to blood sampling
O/027	Streck	Esophageal	254	GOJ. No tumor present, regression from chemotherapy	Original staging was T3 before chemotherapy	R0 ypT0 L0 V0 Pn0 N0 (0/40) M0	None identified	Chemotherapy finished 11 weeks prior to blood sampling
O/028	Streck	Esophageal	273	Tumor at GOJ, size: 30 x 35 mm	T3 N0 M0 at time of bloods. T3 N3 M0 after surgery	N/A	None identified	N/A
P/009	Streck	Pancreatic	243	Distal pancreas. Tumor size: 20 mm	T1 N0 Mx	N/A	None identified	N/A

Table S3: Clinical information for patients with gastrointestinal carcinoma (*n* = 15) continued.

FLOT, Combination of chemotherapy drugs: docetaxel, oxaliplatin, 5-fluorouracil and leucovorin; GOJ, gastro-esophageal junction

Patient ID	Blood collection tube	Cancer type	Platelet count (x10^9/L)	Tumor information (location, size)	TNM staging	Identified mutations	Therapy
C/023	Streck	Colorectal	232	Caecum	T4 N0 M0	Loss of *MLH1,PMS2* IHC staining	Surgery and curative right hemicolectomy
C/026	EDTA	Colorectal	207	Rectum	T4a N1 M0	*KRAS* c.35G>A p.(Gly12Asp)	Surgery & neoadjuvant chemoradiotherapy
O/020	EDTA	Esophageal	249	Distal oesophagus	T2 N1 M0	None identified	Surgery and neoadjuvant chemoradiotherapy
O/021	EDTA	Esophageal	199	Distal oesophagus	T2 N0 M0	None identified	Surgery and neoadjuvant chemotherapy
O/022	EDTA	Esophageal	304	Gastric antrum	T4a N0 M0	None identified	Surgery
P/008*	Streck	Pancreatic	266	Large 11 cm mass involving pancreatic tail, spleen, stomach and transverse colon	T4 N1 M0	None identified	No therapy prior to blood sampling
P/017	Streck	Pancreatic	205	Pancreatic ductal adenocarcinoma	pT3 N1 (2/3) L1V1	None identified	Curative intent surgery
Combat CRC 008	Streck	Colorectal	247	Colon (ascending)	T3 N2 M0	Loss of *MSH2* and *MSH6*, *KRAS* c.38G>A p.(Gly13Asp) detected in the transverse tumor	Unchecked
Combat CRC 009	Streck	Colorectal	330	Colon (hepatic flexure)	T3 N0 M0	MMR immunohistochemistry not performed	Unchecked
Combat CRC 010	Streck	Colorectal	235	Colon (ascending)	T3 N0 M0	Loss of *MLH1* and *PMS2*, *PIK3CA* c.3140A>G p.(His1047Arf)	Unchecked
Combat CRC 011	Streck	Colorectal	150	Colon (sigmoid)	Unavailable	*KRAS* c.183A>Tp.(Gln61His), *TP53* c.1024C>T p.(Arg342)	Neo-adjuvant chemotherapy (oxaliplatin and capecitabine)
Combat CRC 012**	Streck	Colorectal	346	Colon (sigmoid)	T3 N0 M0	*NRAS* codon 12 variant detected	Unchecked
Combat CRC 013	Streck	Colorectal	460	Caecum	T3 N2 M0	*KRAS* variant	Unchecked

Table S4: Clinical information for patients with gastrointestinal carcinoma used for whole genome sequencing study (*n* = 13).

APER, Abdomino Perineal Excision of Rectum; cm, centimeter; FLOT, Combination of chemotherapy drugs: docetaxel, oxaliplatin, 5-fluorouracil and leucovorin;
R-CHOP, Combination of chemotherapy: riituximab, cyclophosphamide, hydroxydaunorubicin hydrochloride (doxorubicin hydrochloride), vincristine (Oncovin) and prednisone;
MMR, mismatch repair

*Copy number variations were detected in the cfDNA and s-pDNA for this patient

** Sample used for targeted mutational analysis

Patient ID	Blood collection tube	Age (years)	Gestation (weeks + days)	Neonatal sex
AS/001	Streck	40	39 + 0	M
AS/002	Streck	32	39 + 3	F
AS/003	Streck	34	39 + 1	M
AS/004	Streck	34	40 + 4	F
AS/005	Streck	24	39 + 6	M
AS/006	Streck	39	40 + 4	F
AS/007	Streck	29	39 + 4	F
AS/008	Streck	32	39 + 2	M
AS/009	Streck	34	41 + 5	M
AS/010	Streck	32	35 + 4	M
AS/011	Streck	34	40 + 0	F
AS/012	Streck	33	35 + 1	F
AS/013	Streck	47	38 + 2	M
AS/014	Streck	40	39 + 0	M
AS/015	Streck	37	39 + 3	F
AS/016	Streck	40	39 + 0	M
AS/017	Streck	33	39 + 0	F
AS/018	Streck	29	41 + 3	F
AS/019	Streck	34	39 + 0	M
AS/020	Streck	34	39 + 0	M
PN/024	Streck	31	PD + 1	M
PN/025	Streck	36	PD + 2	M
PN/026	Streck	33	PD + 1	M
PN/027	Streck	31	PD + 1	F
PN/028	Streck	32	PD + 2	M
PN/029	Streck	40	PD + 1	M
PN/030	Streck	32	PD + 1	M
PN/031	Streck	34	PD + 1	M
PN/032	Streck	24	PD + 1	M
PN/033	Streck	31	PD + 2	M

Table S5: Clinical information for obstetric cohort (*n* = 30).

AS, antenatal sample; F, female; M, male; PD, post-delivery; PN, postnatal

Patient ID	Blood collection tube	Platelet count (x10^9/L)
ITP/001	Streck	3
ITP/002	Streck	490
ITP/003	Streck	18
ITP/004	Streck	31
ITP/005	Streck	157
ITP/007	Streck	35
ITP/008	Streck	34
ITP/014	Streck	80
ITP/015	Streck	17
ITP/016	Streck	21
ITP/017	Streck	42
ITP/018	Streck	231
ITP/020	Streck	12
ITP/021	Streck	76
ITP/022	Streck	10
ITP/023	Streck	2
ITP/024	Streck	18
ITP/025	Streck	27
ITP/026	Streck	256
ITP/027	Streck	52
ITP/028	Streck	375
ITP/029	Streck	465
ITP/030	Streck	335

Table S6: Clinical information for the immune thrombocytopenic purpura patients and controls cohort (n = 23).

ITP, immune thrombocytopenic purpura

Flow Cytometry					
**Nucleic acid stain or antibody**	**Clone**	**Conjugate**	**Dilution**	**Company**	**Catalog number**
CD42b	HIP1	FITC	1:20	BioLegend‡	303903
CD42	HIP1	PeCy7	1:20	BioLegend‡	303916
CD41	HIP8	PE-Cyanine7	1:30	BioLegend‡	303718
CD3	SK7	PE	1:20	BioLegend‡	344805
CD3	OKT3	BV421	1:20	BioLegend‡	317344
CD11b	ICRF44	APC	1:20	BioLegend‡	301309
CD33	WM53	APC	1:20	BioLegend‡	303407
CD19	HIB19	APC-Cyanine7	1:20	BioLegend‡	302218
CD235a	HIR2	BV650	1:20	BD Biosciences§	740583
CD235a	HIR2	FITC	1:20	Invitrogen#	11-9987-82
DAPI (4',6-Diamidino-2-Phenylindole, Dilactate)			1:100*	Invitrogen#	D3571
CD63	H5C6	PB	1:20	BioLegend‡	353012
CD62P	AK4	BV785	1:20	BioLegend‡	304941
SYTO 13 Green Fluorescent Nucleic Acid Stain			1:20	Invitrogen#	S7575
DRAQ5 Fluorescent Probe Solution			1:1200	BioLegend‡	424101
**Microscopy**					
**Nucleic acid stain or antibody**	**Clone**	**Conjugate**	**Dilution**	**Company**	**Catalog number**
SiR-DNA			1:500	Spirochromeˆ	SC007
NUCLEAR-ID Red DNA stain			1:2000	Enzo Life Sciences~	ENZ-2406
DRAQ5 Fluorescent Probe Solution			1:1000	BioLegend‡	424101
Hoechst 33342, Trihydrochloride Trihydrate			1:1000	Invitrogen#	H3570
Primary mouse anti-human CD63	MX-49.129.5	Unconjugated	1:50	Abcam†	ab193349

Table S7: Flow cytometry antibodies used for hematopoietic lineage panel, platelet activation panel, analysis of platelet DNA and RNA content and antibodies and nucleic acid stains used for microscopy.

Microscopy					
**Nucleic acid stain or antibody**	**Clone**	**Conjugate**	**Dilution**	**Company**	**Catalog number**
Primary mouse anti-human LAMP2	H4B4	Unconjugated	1:100	Abcam†	ab25631
Primary mouse anti-human CD62P	AK-6	Unconjugated	1:55	Abcam†	ab6632
Primary rabbit anti-human CD81	EPR21916	Unconjugated	1:100	Abcam†	ab219209
Primary rabbit IgG anti-human TOM20 (D8T4N)	Recombinant (D8T4N)	Unconjugated	1:200	Cell signalling technology¦	42406S
Primary anti-β-Tubulin mouse monoclonal	TUB 2.1	Unconjugated	1:130	Sigma Aldrich°	T5201
Primary anti-mouse p53	1C12	Unconjugated	1:2000	Cell signalling technology¦	2524
Secondary goat anti-mouse IgG H&L		AF568	1:500 or 1:1000	Abcam†	ab175473
Secondary goat anti-rabbit IgG H&L		AF488	1:500	Cell signalling technology¦	4412
Secondary donkey anti-mouse IgG H&L		AF405	1:1000	Abcam†	ab175658
Secondary horse anti-mouse IgG		HRP-linked	1:100	Cell signalling technology¦	7076

Table S7: Flow cytometry antibodies used for hematopoietic lineage panel, platelet activation panel, analysis of platelet DNA and RNA content and antibodies and nucleic acid stains used for microscopy (continued).

*Viability stain, previously diluted 1:100 from 5 mg/ml stock solution

§Becton, Dickinson and Company, Franklin Lakes, US-NJ

‡BioLegend, San Diego, US-CA

#Invitrogen (Thermo Fisher Scientific Inc.,) Waltham, US-MA

ˆSpirochrome, Switzerland

~Enzo Life Sciences (UK) LTD, UK

†Abcam, Cambridge, UK

¦Cell Signalling Technology, Inc., Danvers, US-MA

°Sigma Aldrich, St. Louis, US-MO

SUPPLEMENTARY MOVIE LEGENDS

Movie S1: 3D render of maternal platelet labeled with β-tubulin (blue), with positive signal for a Y-chromosome fluorescent *in situ* hybridization (FISH) probe (orange).

Movie S2: Live cell imaging video showing uptake of fluorescently labeled DNA (magenta) from a COLO205 cell by a platelet from a healthy donor *in vitro*.

Movie S3: Live cell imaging video showing multiple platelets (stained with CD42 488 [blue]) showing signal for fluorescently labeled DNA (magenta) derived from COLO205 cells *in vitro*.

Movie S4: 3D render and rotation video of a platelet (stained with CD42 488 [blue]) with adherent extracellular vesicle (EV) (succinimidyl ester [SE], red) containing DNA (DAPI, yellow).

Movie S5: 3D render and rotation video of a platelet (stained with CD42 488 [blue]) with internal extracellular vesicle (EV) (succinimidyl ester [SE], red) containing DNA (DAPI, yellow).

Movie S6: Live cell imaging video of healthy donor platelets (CD42 AF488, cyan) incubated with H2B-mCh HEL cells genetically engineered to express mCherry-tagged histones (magenta). White arrow shows platelet sequestration of tumor cell-derived histones (magenta) and DNA (DAPI, yellow). Scale bar represents 5 µm, imaged using a Zeiss Spinning Disk Confocal with a 63X oil immersion objective.
